# Solvent Oxidation‐Directed Synthesis of Highly Crystalline Iron Oxide Nanoparticles With Near‐Bulk Magnetization

**DOI:** 10.1002/advs.76632

**Published:** 2026-07-20

**Authors:** Lukas Hertle, Valentin Gantenbein, Joaquim Llacer‐Wintle, Mingchen Ma, Ziyu Li, Pu Luo, Zhengwei Tan, Fei Chen, Chen Chen, Chuanyi Wu, Chenxin Gao, Chenyi Zhou, Carlos Franco, Pere Bruna, Josep Puigmartí‐Luis, Xiang‐Zhong Chen, Bradley J. Nelson, Salvador Pané

**Affiliations:** ^1^ Multi‐Scale Robotics Lab, Institute of Robotics and Intelligent Systems ETH Zürich Zurich Switzerland; ^2^ International Institute for Intelligent Nanorobots and Nanosystems, College of Intelligent Robotics and Advanced Manufacturing, State Key Laboratory of Photovoltaic Science and Technology, Shanghai Frontiers Science Research Base of Intelligent Optoelectronics and Perception, Institute of Optoelectronics Fudan University Shanghai China; ^3^ Zhejiang Key Laboratory of Extreme Environment Functional Materials Yiwu Research Institute of Fudan University Yiwu China; ^4^ College of Physics and Electronic Information Engineering Zhejiang Normal University, Zhejiang Institute of Photoelectronics & Zhejiang Institute for Advanced Light Source Jinhua 321004 China; ^5^ Departament de Física BarcelonaTech (UPC) Institut De Tècniques Energètiques (INTE) Barcelona Research Center in Multiscale Science and Engineering Universitat Politècnica de Catalunya Barcelona Spain; ^6^ Departament de Ciència dels Materials i Química Física Institut De Química Teòrica i Computacional University of Barcelona Barcelona Spain; ^7^ Institució Catalana de Recerca i Estudis Avançats (ICREA), Pg. Lluís Companys 23 Barcelona Spain

**Keywords:** iron oxide, magnetic nanoparticle, nanoparticle synthesis, saturation magnetization, thermal decomposition

## Abstract

Achieving iron oxide nanoparticles with well‐defined morphology and near‐bulk saturation magnetization remains challenging. Here, we report a simple Fe(acac)_3_‐based thermal decomposition strategy that produces highly crystalline Fe_3_O_4_ nanoparticles with tunable sizes and near‐bulk magnetization. We identify solvent oxidation as the critical parameter governing this performance. We reveal that the polar oxygenated species generated during controlled oxidation of the solvent benzyl ether (BE) can coordinate to Fe centers and form new intermediate complexes immediately upon simple mixing. These complexes can alter nucleation–growth kinetics, reducing nucleation density while accelerating particle growth, thereby enabling size control without compromising crystallinity. Consequently, the resulting nanoparticles maintain high crystalline quality and magnetite‐rich stoichiometry, accounting for their enhanced magnetic performance. Moreover, the strategy is compatible with conventional facet‐tuning methods and extendable to doped ferrite systems. Unlike iron oleate routes, which often yield non‐stoichiometric particles with reduced magnetization, or systems requiring complex surfactant engineering, our approach achieves high magnetization through straightforward solvent oxidation control. This work provides a simplified strategy for achieving structurally uniform, magnetically optimized iron oxide nanocrystals.

## Graphical abstract

Controlled oxidation of benzyl ether is established as a key synthetic parameter in the thermal decomposition of iron(III) acetylacetonate. Oxygenated solvent species alter precursor coordination and nucleation–growth kinetics, producing highly crystalline, magnetite‐rich nanoparticles with tunable size, controlled morphology, and near‐bulk magnetization, while remaining compatible with facet engineering and doped ferrite synthesis.

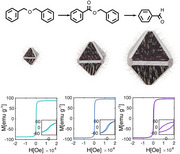

## 1 Introduction

Magnetic iron oxide nanoparticles (MIONPs), primarily magnetite (Fe_3_O_4_) and maghemite (γ‐Fe_2_O_3_), are widely employed in biomedical imaging and therapy due to their high saturation magnetization (M_s_) and biocompatibility characteristics [1, 2, 3, 4]. In applications such as magnetic resonance imaging (MRI) [5, 6, 7], magnetic hyperthermia [8, 9, 10, 11, 12], and targeted drug delivery [13, 14, 15, 16, 17, 18], precise size and shape control together with a high and uniform magnetic response are essential. For example, MRI contrast depends on relaxivity influenced by M_s_, hyperthermia efficiency is highly sensitive to magnetic anisotropy and magnetic losses, and magnetic targeting relies directly on the magnetic force exerted on particles. These magnetic performance metrics are strongly governed by particle size and size distribution, morphology, crystalline quality, and phase composition, motivating the development of simple, scalable, and phase‐pure IONP synthesis strategies capable of delivering well‐defined morphology with high magnetic response. However, achieving such a strategy remains challenging.

Thermal decomposition‐based synthesis has emerged as one of the most effective methods for fabricating monodisperse MIONPs with tunable shapes [19, 20, 21, 22, 23]. Yet geometric control alone is often insufficient to achieve optimal magnetic performance. Increasing evidence suggests that phase purity, stoichiometry (Fe^2+^/Fe^3+^ ratio), and crystalline quality can play a decisive role in determining magnetic properties such as Ms. This is exemplified by iron oleate‐based thermal decomposition syntheses, which can yield particles with well‐defined morphology and narrow size distributions but are frequently associated with mixed phases and non‐stoichiometry, leading to reduced magnetization [24, 25, 26, 27, 28, 29, 30, 31, 32, 33, 34, 35, 36, 37, 38].

Here, we develop a simple and unified Fe(acac)_3_‐based thermal decomposition strategy to synthesize a series of well‐controlled IONPs spanning a broad size range while maintaining high and comparable crystal quality, achieving near‐bulk saturation magnetization (Ms > 80 emu g^−^^1^). One‐pot thermal decomposition of Fe(acac)_3_, offers a straightforward route to highly crystalline, phase‐pure nanoparticles while suppressing reductive side reactions [39, 40, 41, 42]. However, obtaining uniform particle samples of precisely controlled shape requires careful tuning of many parameters, such as solvent polarity, heating rate, precursor concentration, and ligand/additive ratios, whose interactions with nucleation and growth are highly inter‐related. Several studies have attempted to deconvolute these parameter contributions [42, 43, 44, 45, 46, 47]. For example, Qiao et al. demonstrated that oxidation products of benzyl ether (BE), specifically benzaldehyde (BA) and benzyl benzoate (BB), play a critical role in controlling nanoparticle size and dispersity [40]. They also showed that solvent polarity governs particle size (more polar solvents yield smaller cores), while ligand chain length affects shape (shorter ligands favor cubic facets). Nevertheless, their protocol remains relatively complex, involving multiple surfactants and solvents. Muro‐Cruces et al. established a size‐tunable synthesis from 9–80 nm using a simplified recipe, in which a combination of benzyl ether, octadecene, and tetradecene is used along with oleic acid and sodium oleate [39], reducing the synthesis process complexity while making it simultaneously more predictable and controllable.

Inspired by previous studies, we introduce a simplified Fe(acac)_3_‐based thermal decomposition approach in which the degree of benzyl ether oxidation is carefully tuned prior to reaction. Pre‐heating benzyl ether at 60°C for varying durations generates controlled amounts of oxidative byproducts that modulate the iron coordination environment and particle formation kinetics. Combined with existing strategies such as minor adjustments in heating rate, precursor concentration, and the optional addition of sodium oleate, this method allows fine control over particle size and morphology. Notably, the consistently near‐bulk saturation magnetization observed across all samples indicates that high crystallinity and magnetite stoichiometry can outweigh size‐ and surface‐related effects (e.g., dead‐layer‐like behavior) in determining magnetization in our particles. These results demonstrate that pre‐oxidation of the solvent, in combination with a minimal set of reagents, enables the scalable synthesis of structurally uniform and magnetically superior iron oxide nanocrystals across a wide size range.

## 2 Results and Discussion

### 2.1 Influence of Benzyl Ether Oxidation State on the Particle Size and Morphology

**FIGURE 1:**
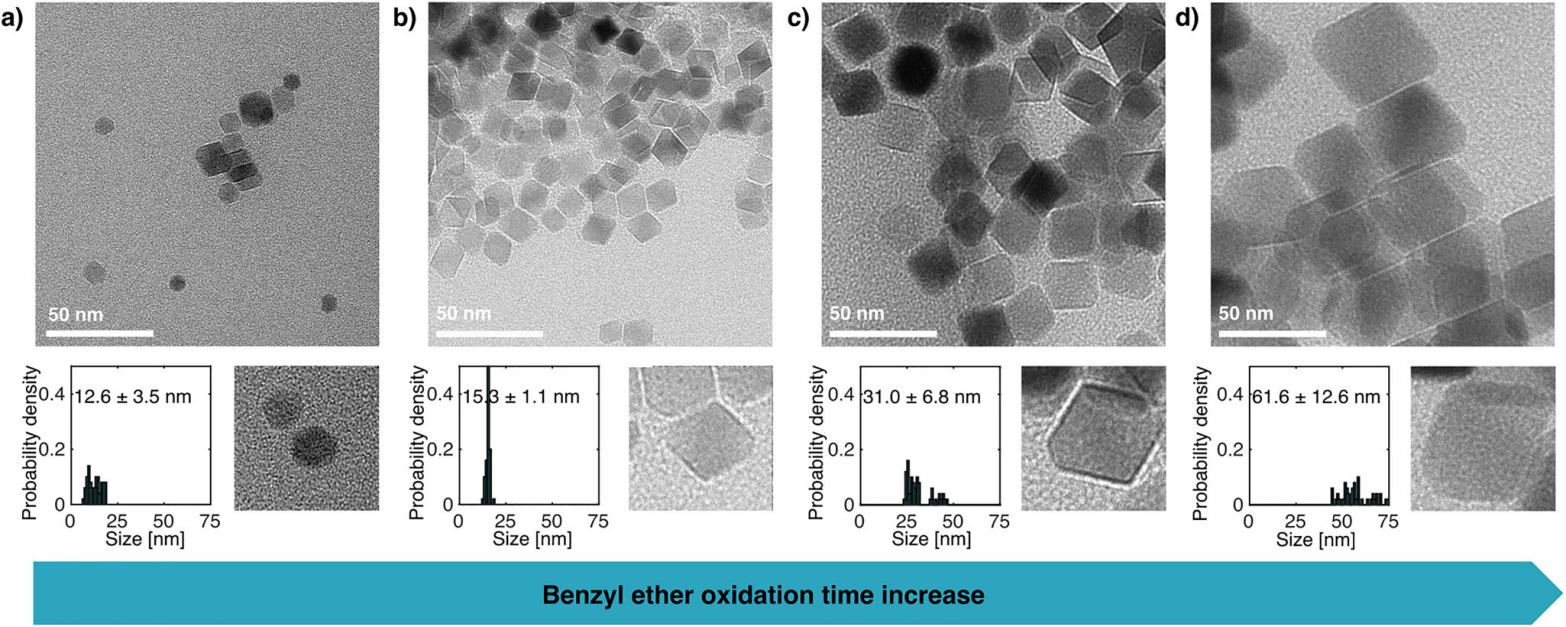
TEM images and size distributions of nanoparticles synthesized with benzyl ether oxidized at 60°C for different durations: (a) 0, (b) 15, (c) 30, and (d) 50 h.

The transmission electron microscopy (TEM) images of nanoparticles synthesized with benzyl ether pre‐oxidized at 60°C for increasing durations (see methods) revealed a progressive growth in particle size (Figure 1a–d and Figure S1). Specifically, samples obtained from BE oxidized for 0 h yielded small, polydisperse particles with irregular geometries (Figure 1a), while a moderate oxidation period of 15 h led to well‐defined octahedral nanocrystals with a narrow size distribution (Figure 1b). Further extending the oxidation duration to 30 and 50 h resulted in particles with significantly larger sizes (Figure 1c,d). Statistical analysis of the particle dimensions, extracted from over 50 particles per sample, quantitatively confirmed this trend. The mean particle size increased from 12.6 ± 3.5 nm (0 h) to 61.6 ± 12.6 nm (50 h), with the standard deviation also expanding at longer oxidation durations.

**FIGURE 2:**
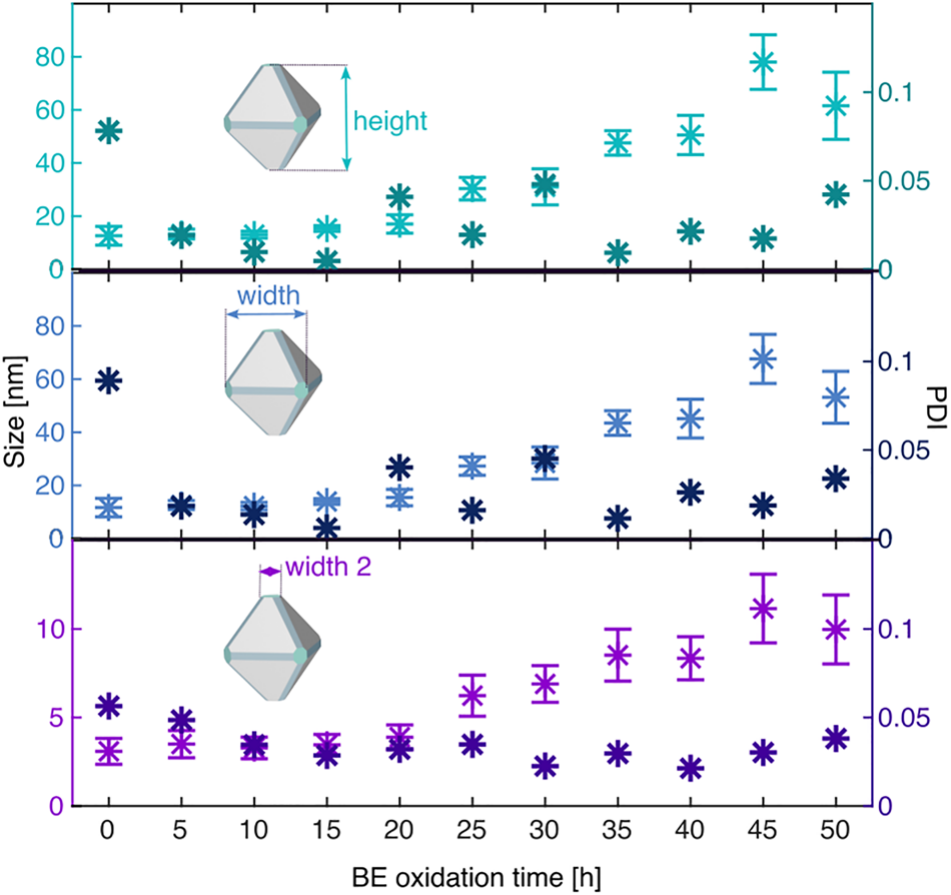
Size distribution and polydispersity index (PDI) of particles synthesized with benzyl ether oxidized for different durations.

To better understand the dimensional evolution, we conducted a shape‐specific morphometric analysis based on the height, width, and edge length of the truncated‐octahedral particles (Figure 2). The data showed that both the particle height and width increased consistently with oxidation time. Notably, the particles retained a quasi‐octahedral geometry throughout the series, as evidenced by consistent aspect ratios between the main dimensions. However, a slight broadening in the distribution at long oxidation times (≥35 h) indicates a tendency toward shape irregularity at elevated oxidative conditions. These results suggest that the oxidative decomposition of BE introduces specific byproducts, likely benzaldehyde and benzoic acid derivatives [40], that modulate the coordination environment of Fe(acac)_3_ and impact the kinetics of nucleation and facet‐selective growth.

### 2.2 Evolution of Solvent Composition During Oxidation

**FIGURE 3:**
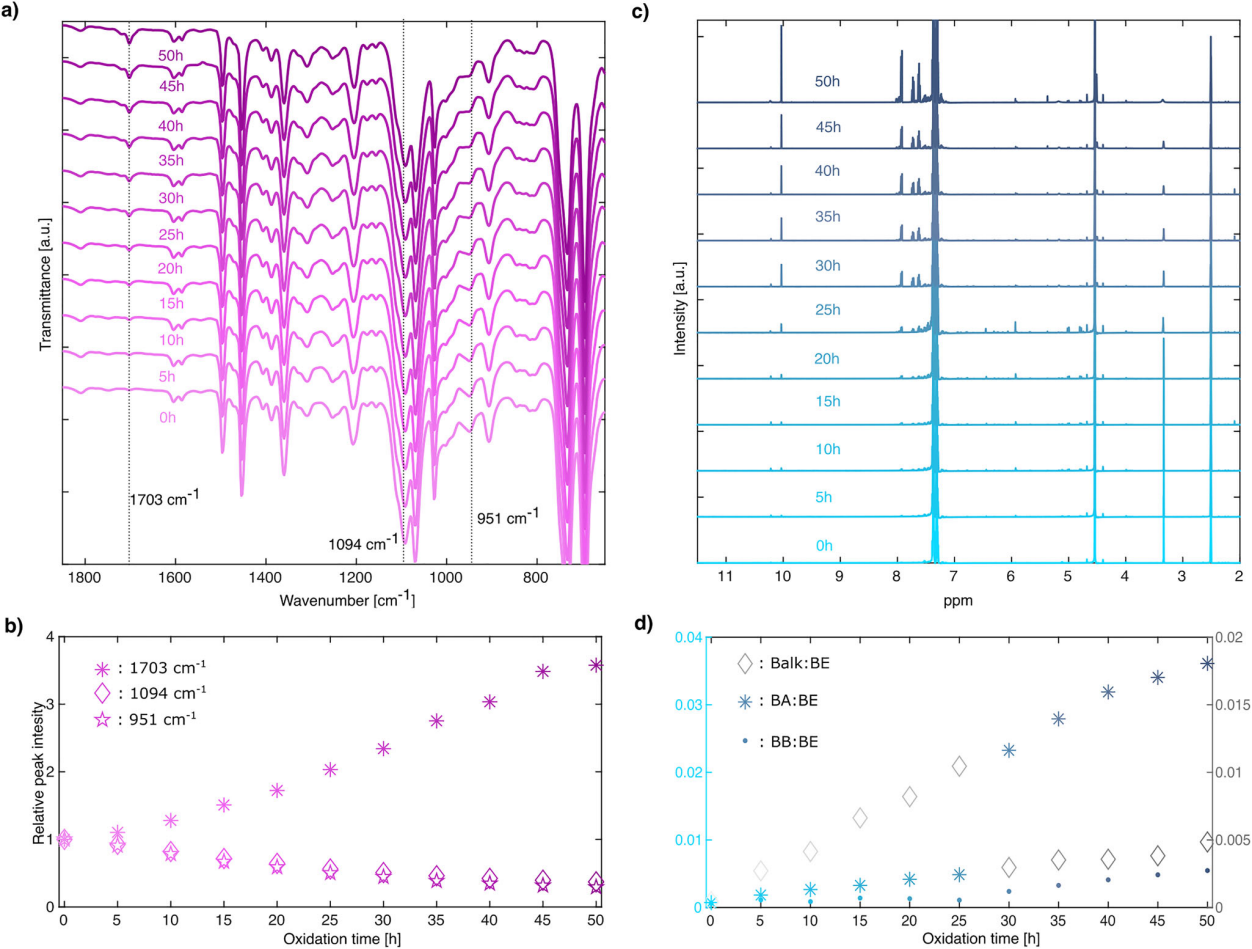
(a) FTIR spectra of benzyl ether oxidized at 60°C for different durations. (b) Changes in the intensity of representative absorbance peaks with increasing oxidation time. (c) NMR spectra of benzyl ether oxidized at 60°C for different durations. (d) The time‐dependent peak integral ratios between peaks associated with Balk (5.93 ppm), BA (10.03 ppm), BB (5.37 ppm) and BE (4.54 ppm).

Fourier transform infrared spectroscopy (FTIR) spectra of benzyl ether oxidized for different durations are presented in Figure 3a and Figure S2. As displayed, a new band at ∼ 1703 cm^−1^, which is characteristic of aromatic C═O (aldehyde/carboxylic) stretching vibration, appears by ∼ 5–10 h, and increases over oxidation time. Meanwhile, the intensity of BE's signature bands near 951 and 1094 cm^−1^, which can be assigned to C─O─C bending and stretching vibration, drops (as is shown in Figure 3b). These results indicate the cleavage of the benzyl–oxygen bond in BE and formation and accumulation of new aromatic species with carbonyl groups, which may be benzaldehyde and benzoate derivatives. Such product formation aligns with prior observations that benzyl ether yields benzaldehyde and benzyl benzoate upon heating [40, 48].

The ^1^H NMR spectra shown in Figure 3c, provide additional evidence of the benzyl ether oxidation products. A singlet at ∼ 5.9 ppm, first gradually increases in intensity and then almost disappears after ∼ 25 h. As displayed in Figure S3, this singlet cannot be associated with the reported oxidation products of benzyl benzoate and benzaldehyde, but most likely corresponds to an alcohol OH group. Quantitative integration of the alcohol (Balk), BB and BE associated peak signals (Figure 3d) shows the Balk/BE ratio rising to ∼ 0.01 around 20 h before a sharp decrease to ∼ 0.003 was observed, followed by a modest increase to 0.005 by 50 h. In contrast, BA appears more slowly: a distinct aldehydic proton at ∼ 10.0 ppm grows steadily over the first 25 h, then jumps and accelerates thereafter (Figure 3d), reaching a BA/BE ratio of ∼ 0.036 by 50 h. The BB associated proton at ∼ 5.4 ppm, followed a similar trend, however, only a maximum ratio of BB/BE ∼ 0.0055 was observed after 50 h of oxidation. Together, these trends indicate that BE oxidation initially leads to the formation of an alcohol, which, after accumulating, results predominantly in the formation of BA, accompanied by a small amount of BB. This oxidation trend is consistent with previous reports, which describe the formation of BA following the initial formation of an alcohol derivative from BE [49]. Overall, the NMR data therefore confirm a stepwise oxidation pathway: BE → Balk → BB + BA, followed by oxidation of BB to BA. Interestingly, the originally used BE contained a notable amount of water, which diminishes as oxidation proceeds (especially after ∼25 h). We hypothesize that this loss of water during shorter oxidation times contributes at least in part to the observed improvement in nanoparticle uniformity.

The infrared and NMR spectra clearly proved that, over extended heating, the solvent evolves into a mixture rich in polar oxidation products such as benzyl benzoate and benzaldehyde, which can coordinate Fe(III) much more strongly than BE, and alter precursor speciation, which is likely to modulate nucleation kinetics and particle uniformity compared to pristine BE.

### 2.3 Oxidized Products Modulate Coordination State of the Precursor and Reaction Process

**FIGURE 4:**
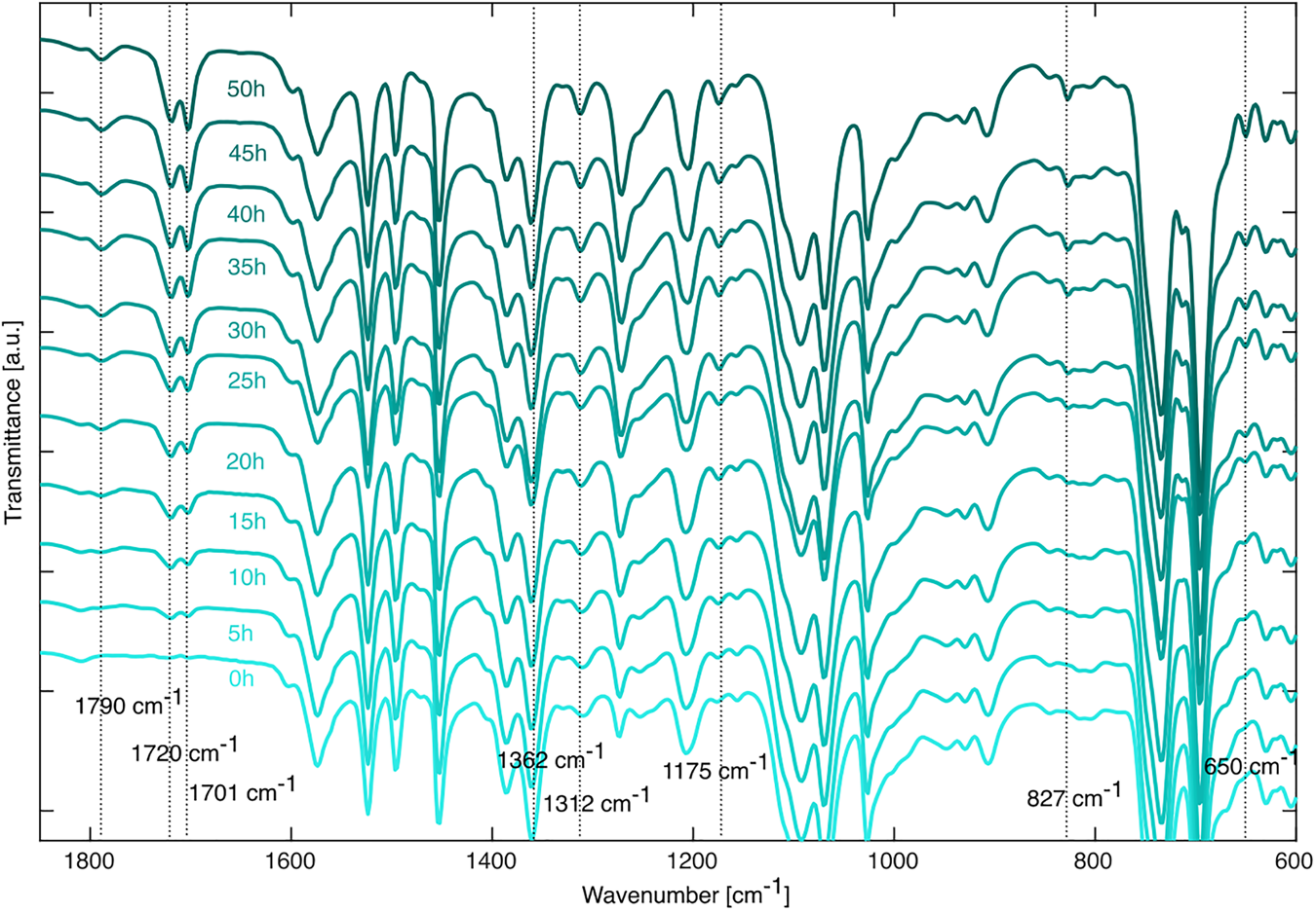
FTIR spectra of the mixture of Fe(acac)_3_ and BE pre‐oxidized for different durations. The mixtures were prepared at room temperature by mixing the two reagents via strong agitation for 30 s.

Upon mixing differently oxidized benzyl ether (BE) samples with iron(III) acetylacetonate (Fe(acac)_3_), the FTIR spectra (Figure 4 and Figure S4) revealed distinct changes compared to the spectra of the individual components. Specifically, new peaks emerged at 1790, 1720, 1701, 1312, 1175, 827, and 650 cm^−1^. These spectral features became increasingly pronounced with longer oxidation times of BE. As depicted in Figure S5, these signals cannot be reproduced by a simple overlay of the spectra from the pure oxidized BE and Fe(acac)_3_, confirming the formation of new chemical species in the mixture.

The new carbonyl‐region bands suggest multiple oxidation and ligand‐exchange processes. For example, the split peaks at 1720 and 1701 cm^−1^ likely arise from further oxidation of benzaldehyde to benzoic acid. Benzaldehyde is known to autoxidize to benzoic acid in air, especially with metal catalysts [50, 51, 52, 53, 54]. Therefore, Fe^3+^ ions could accelerate this conversion, yielding a benzoic acid C═O stretch near 1695–1705 cm^−1^. This may account for the new absorption near 1703 cm^−1^. Alternatively, these peaks may also reflect release of acetylacetone ligands from Fe(acac)_3_. As benzaldehyde and benzoic acid bind strongly to iron, they can displace acetylacetonate, freeing acetylacetone whose C═O stretch appears in this region. The unusual peak at ∼ 1790 cm^−1^ is particularly informative. It does not match any known vibration of benzaldehyde, benzoic acid, or benzyl benzoate, but is characteristic of highly conjugated or strained carbonyls such as anhydrides (acid anhydrides typically show C═O stretches near 1820 and 1760 cm^−1^). The combination of the ∼ 1790 cm^−1^ band and a companion at ∼ 1720 cm^−1^ fits an anhydride‐like vibration. This observation implies that further oxidation may occur when oxidized BE is mixed with Fe(acac)_3_, potentially forming benzoic anhydrides or related species. At the same time, Fe(acac)_3_ appears to undergo partial ligand exchange with the oxidized BE components as suggested by the slight decrease in intensity of the characteristic Fe(acac)_3_ peak near ∼1362 cm^−1^, which indicates the dissociation of acetylacetonate and coordination of benzaldehyde/benzoic acid to the iron center.

These findings highlight that the solvent is not merely a passive medium. Oxidized BE species (benzaldehyde, benzoic acid, etc.) interact directly with the iron precursor, altering its coordination environment even at room temperature. As a result, the nanoparticle formation pathway depends on the oxidation state of BE, leading to different in situ metal complexes during heating. In other words, varying the BE oxidation state modulates the nucleation chemistry of the iron precursor.

**FIGURE 5:**
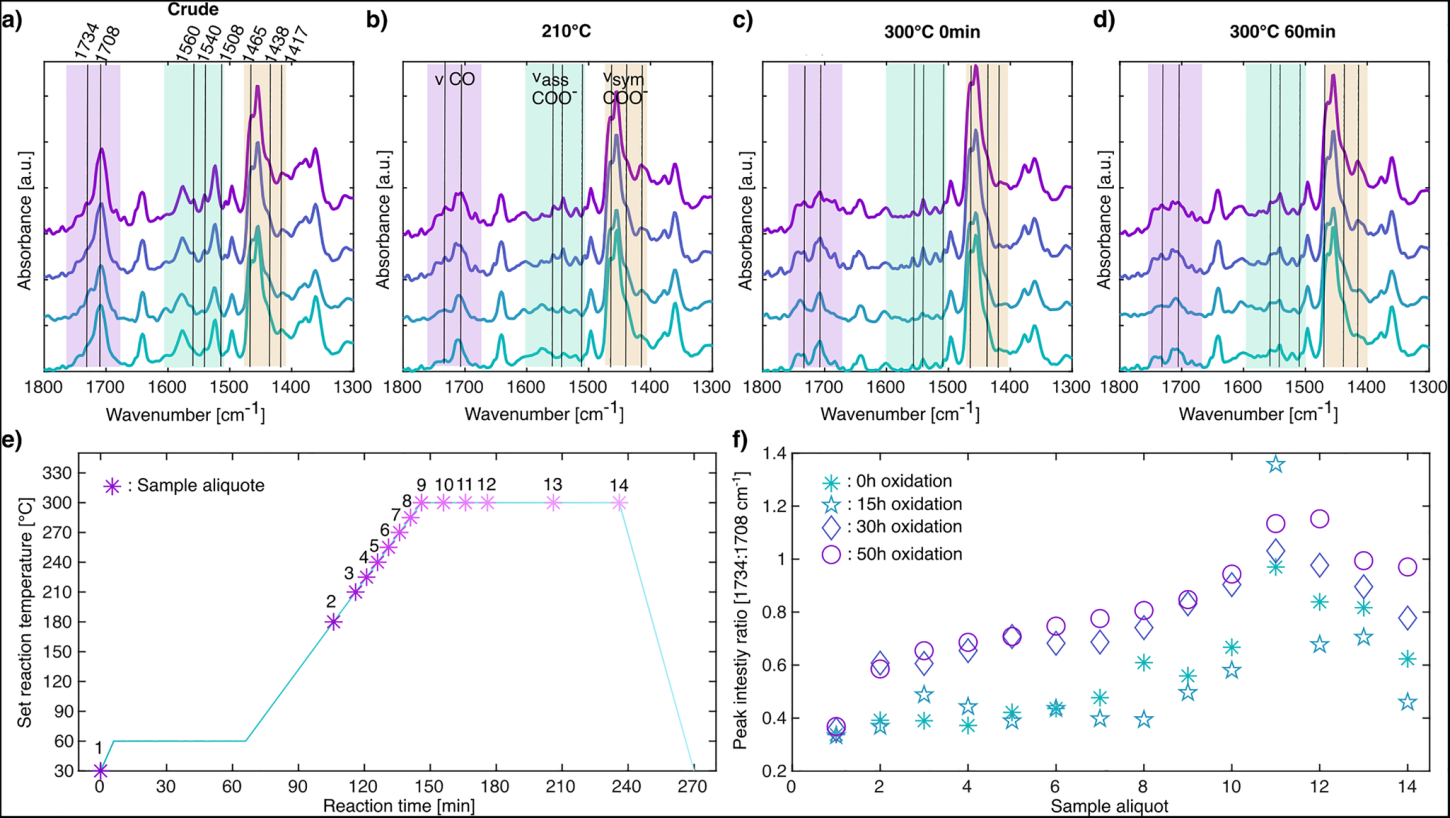
FTIR spectra of reaction mixture samples taken throughout different times of the synthesis (from bottom to top: 5, 15, 30, and 50 h). (a) Crude reaction mixture, (b) mixture at 210°C, (c) mixture at reflux for 0 min, (d) mixture at reflux for 60 min. (e) Conceptual illustration of temperature profile of the reaction and time steps at which sample aliquots were taken. (f) Peak intensity ratio between 1734 and 1708 cm^−1^ peaks for sample aliquots taken throughout the different synthesis.

To investigate the evolution of the iron precursor in differently oxidized BE, we conducted ex situ FTIR analyses of the reaction mixtures by taking sample aliquots at different times during the synthesis (Figure 5a–d and Figure S6). In the crude (unheated) samples, peaks at 1540 and 1560 cm^−1^ emerge only for BE oxidized for 15 h, 30 h or 50 h. In contrast, they are absent in mixtures made with non‐oxidized BE. The 1650–1500 cm^−1^ region corresponds to the asymmetric stretch of coordinated carboxylates, while the 1400–1450 cm^−1^ region corresponds to symmetric stretches. The separation Δν = ν_asym—_ν_sym_ indicates coordination mode: Δν > 200 cm^−1^ implies monodentate binding, Δν < 110 cm^−1^ suggests bidentate binding, while intermediate values indicate bridging coordination. The presence of the 1540 and 1560 cm^−1^ peaks in the more strongly oxidized samples indicates a greater extent of bidentate carboxylate binding compared to the less oxidized BE samples. Previous studies have shown that stronger bidentate coordination stabilizes larger iron oleate clusters during nucleation, correlating with the formation of larger nanoparticles.

Moreover, the evolution of oleic acid coordination bands differs with BE oxidation. In mixtures from more oxidized BE, the FTIR band at ∼ 1734 cm^−1^ (unidentate‐bound oleic acid C═O) appears earlier and grows more rapidly than in less oxidized systems, whereas the free oleic acid C═O band at ∼ 1708 cm^−1^ diminishes more quickly (Figure 5e,f). This shift suggests that oxidized solvents accelerate the coordination of oleic acid to iron, likely due to changes in precursor speciation and competitive binding from benzaldehyde/benzoic acid.

Taken together, these results indicate that both the strength and timing of iron–carboxylate complex formation depend sensitively on the BE solvent's oxidation state. This mechanistic insight directly connects to the previously reported particle growth behavior: stronger iron‐ligand interactions (as induced by more oxidized BE) are known to favor the formation of larger, more uniform iron oxide nanoparticles [55]. This change in size might also be related to the change in the reaction kinetics [56, 57], as revealed in the ex situ small‐angle X‐ray scattering (SAXS) measurement (Figure S7).

### 2.4 Oxidation Strategy Compatible With Other Morphology‐Control Strategies and Ferrite Types

**FIGURE 6:**
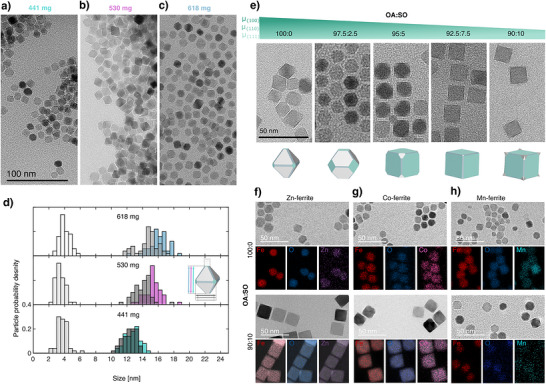
TEM images (a–c) and size distribution (d) of particles synthesized with different amounts of iron(III) acetylacetonate using BE oxidized for 15 h. (e) TEM images of particles synthesized with different amounts of oleic acid and sodium oleate using BE oxidized for 15 h. (f–h) TEM images and EDX maps of ferrite particles of different morphologies synthesized with BE oxidized for 15 h.

We also explored whether the oxidation process could influence the size‐ and shape‐tuning strategies previously developed by other groups. Specifically, we investigated the impact of varying the metal precursor concentration on nanoparticle size. By carefully adjusting the amount of precursor, we were able to modify the particle size from approximately 12.7 ± 0.9 nm to 16.1 ± 1.1 nm (Figure 6a–d), while maintaining consistent morphology. Furthermore, we attempted to fine‐tune particle morphology by suppressing {100} and {110} facet growth through the introduction of sodium oleate into the reaction. Figure 6e and Figure S8 illustrate the evolution of nanoparticle shape as the ratio of oleic acid to sodium oleate was varied, while keeping the total oleate concentration constant.

The resulting particles transitioned from an octahedral to a tetradecahedral and then to a cubic morphology, ultimately reaching a kinetically trapped, slightly octopodal shape, while keeping their average size ∼ 15 nm. We also extended this strategy to the synthesis of other ferrites, such as Zn‐ferrite, Co‐ferrite, and Mn‐ferrite nanoparticles with the desired morphology (Figure 6f–h). EDX maps confirmed the particle compositions close to the precursor feeding ratio of 1:2 for Co‐ and Mn‐ferrite but deviated for Zn‐ferrite (feeding ratio 0.6:2.4, detected ratio 0.27:2.73 for Zn:Fe). The discrepancy may be due to the fact that the metal oleates formed in situ have different decomposition profiles. Zn‐oleate usually has higher thermal stability compared to Fe‐oleate, and therefore Zn‐oleate and Fe‐oleate decompose asynchronously, leading to deviation in the final composition. In contrast, Co and Mn are known to form mixed‐metal oleate precursors, which allow for the formation of trinuclear oxo‐clusters containing both metal ions, enabling homogeneous growth of the metal‐ferrite nanoparticles [58]. All these results indicate that the presence of oxidized products in benzyl ether did not interfere with the existing particle tuning strategies, indicating that the oxidation method can be effectively combined with size and morphology control techniques. This suggests that our oxidation strategy can be integrated with other simple methods to further tune particle size, enhancing the potential for controlled nanoparticle synthesis in various applications.

### 2.5 Influence of Oxidation Time on the Structure, Magnetic Properties and Colloidal Stability of Particles

**FIGURE 7:**
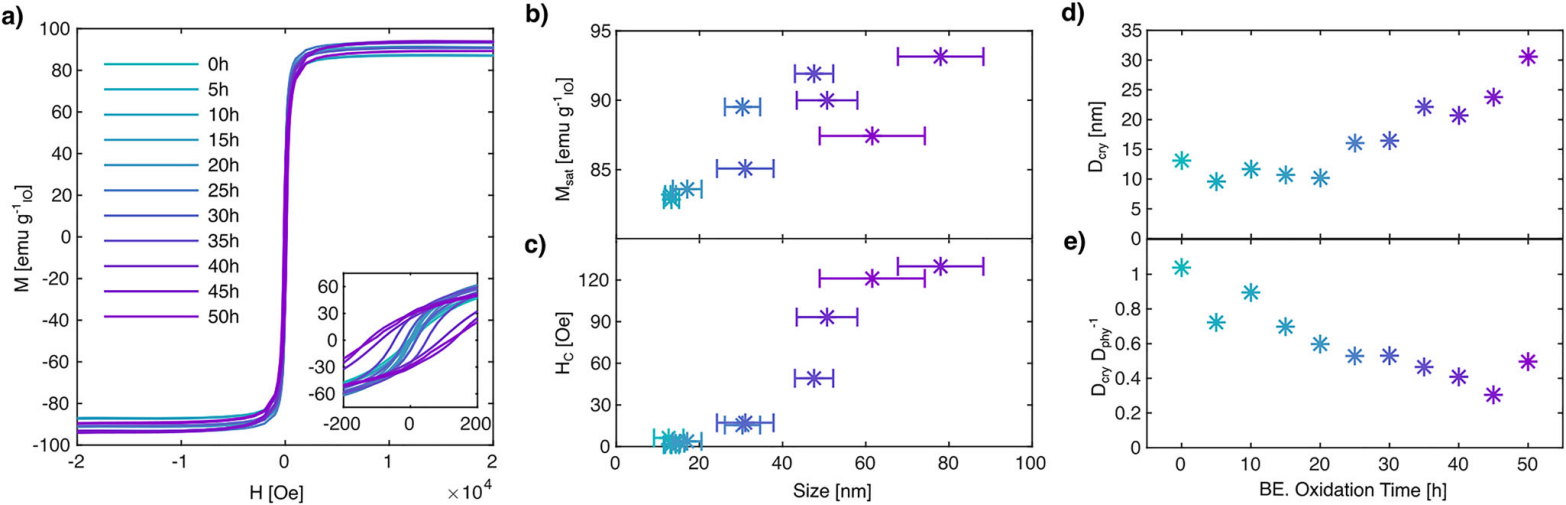
(a) Hysteresis loops of nanoparticles synthesized with BE oxidized for different durations. Size‐dependent saturation magnetization, (b) and coercivity, (c) of nanoparticles. Size of crystallites, (d) and their ratio to the average height of the nanoparticles in dependency of the oxidation time of BE.

Magnetic hysteresis loops at 300 K (Figure 7a,b) show a gradual increase in saturation magnetization M_s_ from 83 to 94 emu/g_IO_ and an accompanying rise in coercivity H_c_ with increasing benzyl ether (BE) oxidation time. This trend clearly correlates with the increase in particle size, which is determined by the oxidation time of BE. The enhanced coercivity suggests a transition of the nanoparticles from a superparamagnetic to a ferromagnetic state (Figure 7c) [59]. Additionally, the increase in saturation magnetization may be related to changes in the phase composition of the nanoparticles. Fe^2^^+^ ions are unstable in very small nanoparticles and tend to oxidize to Fe^3^^+^, resulting in the formation of γ‐Fe_2_O_3_, which exhibits slightly lower magnetization (Bulk material Ms: ∼ 72–80 emu/g) [60], compared to pure Fe_3_O_4_ (Bulk material Ms: 90–93 emu/g at 300 K) [61, 62]. As particle size increases, Fe^2^^+^ becomes less prone to oxidation, favoring the formation of Fe_3_O_4_, which has a higher saturation magnetization.

X‐ray diffraction (XRD) results show that all samples display typical inverse spinel structures, consistent with magnetite and maghemite phases. Subsequent calculations of the particles’ crystalline diameters using the Scherrer equation, based on the FWHM obtained from a Gaussian fit of the MIONPs’ (311) peak, confirmed the expected increase in crystal size with increasing BE oxidation time (Figure 7d). However, a direct comparison between the calculated crystal size and the physical sizes of the particles revealed a progressive decrease in their correlation as BE oxidation time increased (Figure 7e). This trend may be attributed to the formation of a multi‐domain configuration within the MIONPs, as the critical size for maintaining a single‐domain state in iron oxide nanoparticles is approximately 25–30 nm, or to increasing internal microstrain (Figure S9). Careful inspection of diffraction peak positions additionally indicates a slight shift toward lower 2θ values with prolonged oxidation (Figure S10), suggesting a decreasing presence of maghemite [63]. Rietveld refinement further confirmed this trend (Figures S10 and S11). Magnetic characterizations of size and morphology tuned particles were further conducted, which only revealed modest changes in their magnetic response in dependency of the underlying particle parameters (Figure S12).

**FIGURE 8:**
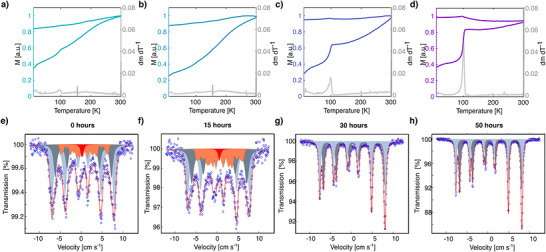
Zero‐field cooling and field cooling (ZFC‐FC) curves with the derivatives of ZFC magnetization (grey line of samples: a) 0 h, b) 15 h, c) 30 h, d) 50 h. Room temperature Mössbauer spectra of particles synthesized with BE oxidized for different times. The blue points represent the experimental data, the red‐line the total fit and the shaded area the four subspectral components (orange: distribution; light grey Fe^3+^; dark grey Fe^2.5+^; red: superparamagnetic) (e) 0, (f) 15, (g) 30, and (h) 50 h.

To verify the correlation between the increasing amount of magnetite in the particles and the increasing solvent oxidation time, we further assessed the temperature‐dependent magnetic response and the Mössbauer spectroscopy. The magnetic response under zero‐field‐cooling and field‐cooling (ZFC‐FC) conditions (Figure 8a–d) revealed a clear sharpening of the Verwey transition (as indicated by the ZFC derivative), which is the fingerprint of magnetite, confirming an increasing Fe^2^^+^/Fe^3^^+^ ratio in MIONPs synthesized with longer BE oxidation times. Figure 8e–h and Table S1, present the Mössbauer spectra with their respective hyperfine parameters. For the 30 and 50 h samples, two sextets were sufficient to obtain good fits, with hyperfine parameters corresponding to Fe^3^^+^ in tetrahedral sites and mixed‐valence Fe^2^^+^/Fe^3^^+^ in octahedral sites (denoted Fe^2^·^5^^+^, as Mössbauer spectroscopy at room temperature cannot resolve Fe^2^^+^ and Fe^3^^+^ separately in octahedral sites, we estimated their ratio by assuming equal populations of Fe^2^^+^ and Fe^3^^+^). For the 15 h and 0 h samples, broader peak widths required the inclusion of a hyperfine magnetic field distribution (between 10 and 37 T) to fit the spectra accurately. These distributed components are generally attributed to surface Fe atoms with lower crystallinity [64, 65], consistent with the increased surface‐to‐volume ratio in smaller particles. Additionally, a doublet was included to represent superparamagnetic particles below the critical size threshold. As shown in Table 1, a clear increase in Fe^2^^+^ content was observed with increasing BE oxidation time, correlating well with the increase in magnetic saturation at 300 K.

**TABLE 1:** Resonant absorption area (at%) for divalent and trivalent Fe atoms of the spinel structure assuming an equal distribution of the two cations in the octahedral sites together with their ratio S (Fe^2+^/Fe^3+^).

	0 h	15 h	30 h	50 h
Fe^3+^	70.2 (6)	69.7 (6)	74.3 (6)	71.3 (6)
Fe^2+^	13.5 (3)	16.7 (3)	25.7 (3)	28.7 (3)
S	0.19 (1)	0.28 (1)	0.35 (2)	0.40 (1)

In summary, longer BE oxidation (larger particle size) produces NPs with higher Ms and H_c_, reflecting reduced surface disorder and higher Fe^2^^+^ content. The data imply that small NPs tend to oxidize to γ‐Fe_2_O_3_ (maghemite) with lower magnetization, while larger NPs retain more Fe^2^^+^ (magnetite), as evidenced by the Mössbauer spectroscopy.

It has been debated whether the magnetization of iron oxide nanoparticles decreases significantly below 20 nm in size. This reduction is often attributed to a so‐called “magnetic dead layer,” which is characterized by surface spin disorder caused by dangling bonds, amorphous region on the surface, and so on [66, 67, 68, 69]. However, this explanation may be overstated, as the magnetic dead layer is typically very thin, often less than 1 nm, and cannot fully account for the observed drop in saturation magnetization, especially for the particles over 10 nm. In our case, all the samples are over 10 nm. Therefore, we believe that the intrinsic crystalline quality of the nanoparticles plays a more critical role compared to the surface effect on the magnetic properties. Factors such as phase composition, high structural quality (low defect density), and an optimal Fe^2^^+^/Fe^3^^+^ ratio (stoichiometry) are the primary determinants of strong magnetic performance. Furthermore, smaller particles are more prone to Fe^2^^+^ oxidation, resulting in a higher proportion of γ‐Fe_2_O_3_ and, consequently, lower magnetization. In this study, we synthesized a series of high‐crystallinity nanoparticles with near‐bulk saturation magnetization, which clearly demonstrated this size‐dependent trend.

**FIGURE 9:**
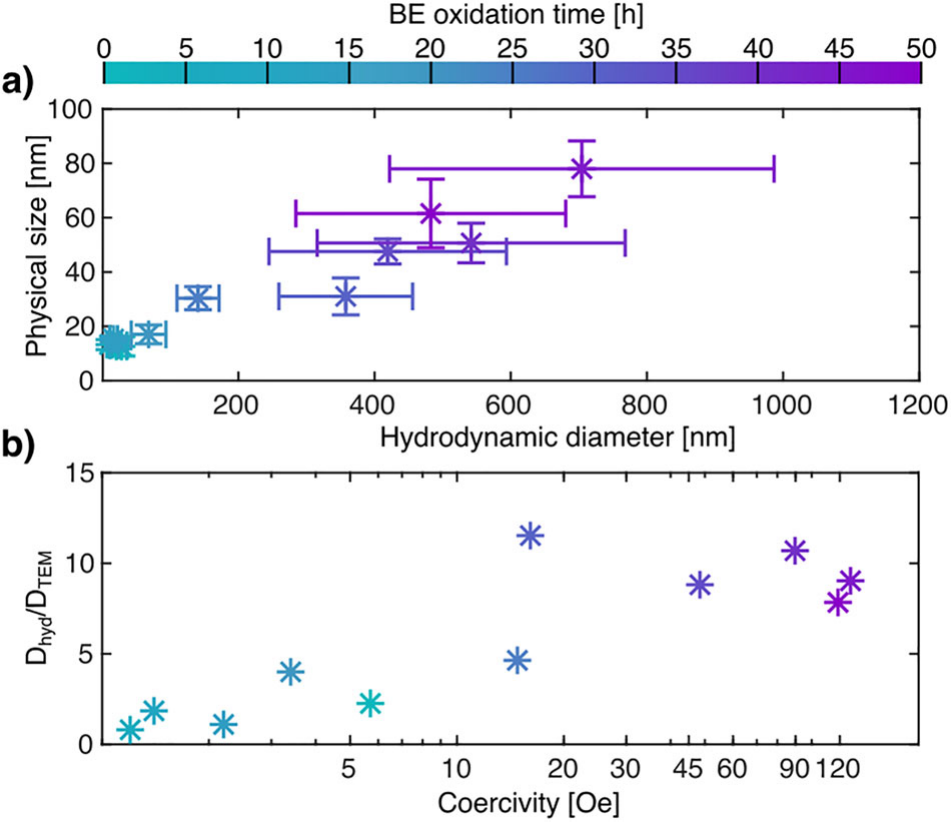
Hydrodynamic diameter of the nanoparticles. (a) Measured hydrodynamic diameter vs. Particle size derived from TEM images of particles synthesized with BE oxidized for different time duration. (b) Hydrodynamic diameter/TEM diameter as a function of the particles magnetic coercivity of nanoparticles synthesized with BE oxidized for different time durations.

Lastly, we investigated the dispersion state of the as‐synthesized particles via dynamic light scattering (DLS). As depicted in Figure 9a, the hydrodynamic diameter (D_hyd_) of the particles increased consistently with the increasing physical diameter obtained from TEM (D_TEM_). Notably, when D_TEM_ is over 20 nm, the D_hyd_ and its standard deviation increase by several orders of magnitude compared to D_TEM_, indicating enhanced aggregation. A clear correlation between D_hyd_/D_TEM_ (which reflects the aggregation state of the particles) and the particles’ magnetic coercivity was observed (Figure 9b), which indicates that the increased aggregation and standard deviation can be attributed to enhanced magnetic dipolar interaction between the MIONPs, as observed also in previous reports [39].

## 3 Conclusions

In this study, we demonstrate that the oxidation state of BE plays a critical role in regulating the growth and magnetic properties of iron oxide nanoparticles synthesized via thermal decomposition of Fe(acac)_3_. By systematically varying the BE oxidation time, we establish a clear correlation between solvent oxidation, particle size, and resulting magnetic behavior. Spectroscopic analyses (FTIR, NMR, Mössbauer) reveal that BE oxidation generates polar oxygenated species such as benzaldehyde and benzoic acid, which modify the coordination environment of the iron precursor, alter reaction kinetics, and influence nanoparticle growth. Importantly, the BE oxidation strategy does not interfere with established size‐ and facet‐control methods, enabling access to a broad range of morphologies and ferrite compositions. Unlike earlier approaches that often rely on changes in reaction conditions, precursor compositions, solvent mixtures, or ligand/additive ratios to tune particle properties, our method uses the controlled oxidation state of benzyl ether as the primary variable while keeping the core reaction formulation and synthesis conditions otherwise unchanged. This provides a more standardized strategy to decouple particle tuning from simultaneous variations in multiple synthesis parameters. It should be noted, however, that the oxidation behavior of benzyl ether can exhibit quantitative variations depending on the solvent batch and supplier. Therefore, preliminary characterization of the oxidation kinetics under the specific experimental conditions employed is recommended to ensure reproducible control over the nanoparticle synthesis process.

Despite variations in size, all nanoparticles exhibit high crystallinity and strong magnetization. Larger particles retain better stoichiometry and phase purity, with higher Fe^2^^+^ content and a M_s_ close to bulk‐Fe_3_O_4_. Smaller particles, with sizes above 12 nm and magnetite‐rich phase, exhibit slightly lower M_s_ value but still higher than 80 emu/g. The results indicate that the crystallinity and stoichiometry are predominant factors in defining the magnetic properties in these particles.

Although the present study focuses on iron oxide nanoparticles, these findings may also be relevant to other colloidal nanocrystals synthesized through thermal decomposition routes, where subtle variations in the solvent composition and reaction environment can influence precursor chemistry, reaction kinetics, and ultimately nanocrystal formation. More broadly, our results suggest that standardized protocols with well‐controlled and well‐characterized reaction parameters are essential for achieving reproducible outcomes in colloidal nanocrystal synthesis. Future work could employ in situ analysis techniques to quantitatively resolve how the oxidation state of the solvent influences nucleation density, precursor‐conversion kinetics, and nanoparticle growth [56, 57, 70, 71, 72].

## 4 Experimental Section/Methods

### 4.1 Benzyl Ether Oxidation

Commercial grade benzyl ether (BE) (Thermo‐Scientific, CAS: 103‐50‐4), was oxidized by vigorously stirring 200 mL of BE in a 500 mL Erlenmeyer flask at 60°C (1200 rpm) under atmospheric conditions. This temperature was shown to be ideal for fine‐tuning the ratio of the formed oxidation products. A lower temperature would significantly elongate the required oxidation time while a higher temperature might induce other reactions. Sample aliquots were taken after pre‐defined time durations and subsequently stored under inert conditions for further processing and analysis.

### 4.2 Particle Synthesis

In a typical nanoparticle synthesis, iron acetylacetonate (530 mg, 1.5 mmol) (Thermo Fisher, CAS: 14024‐18‐1) was dissolved in 7 mL of pre‐oxidized benzyl ether (Thermo Scientific, CAS: 103‐50‐4) by sonication, before adding 15 mL of 1‐octadecene (Sigma‐Aldrich, CAS: 112‐88‐9) and 3 mL of 1‐tetradecene (Sigma‐Aldrich, CAS: 1120‐36‐1). Subsequently, oleic acid (1.854 g, 6.56 mmol) (Sigma‐Aldrich, CAS: 112‐80‐1) was added to the reaction slurry, and the mixture was homogenized again by sonication. The mixture was then degassed for 60 min at 60°C at 0.3 mbar under vigorous stirring before being heated to reflux at 294°C at a heating rate of 3°C min^−^^1^ under a constant flow of N_2_. After refluxing for 90 min (to ensure the reaction completion), the particle‐containing solution was allowed to cool to room temperature before being stored at 4°C for further investigation.

### 4.3 Particle Purification

As synthesized nanoparticles were purified by precipitation via centrifugation after the addition of 20 mL chloroform (Sigma–Aldrich, CAS: 67‐66‐3) and 100 mL acetone (Sigma–Aldrich, CAS: 67‐64‐1) to the crude product. After precipitation, the collected particles were washed two more times by re‐dispersion in 25 mL chloroform followed by the addition of a mixture of 50 mL methanol (Sigma Aldrich, CAS: 67‐56‐1) and 50 mL acetone, before precipitation via centrifugation.

### 4.4 Transmission Electron Microscopy (TEM)

TEM images of the particles were acquired using an FEI Talos F200X (Chem S/TEM) operating at 200 kV, equipped with an X‐FEG emitter and a CETA camera. Sample preparation was carried out by drop‐casting purified nanoparticles dispersed in chloroform onto a carbon film‐coated copper grid. Size distribution analysis was performed by manually measuring three different diameters of over 50 individual nanoparticles using FIJI (ImageJ) software.

### 4.5 Fourier Transform Infrared Spectroscopy (FTIR)

FTIR spectra of the different samples were taken either with a Bruker Tensor 27 equipped with a Platinum ATR, or a Varian 640 Fourier Transform Infrared Spectrometer equipped with a Golden Gate diamond ATR. Sample spectra were recorded using either 16 or 200 scans in the range of 4000–600 cm^−1^, with a step size of 4 cm^−1^.

### 4.6 Nuclear Magnetic Resonance Spectroscopy (NMR)

^1^H NMR and ^13^C NMR spectra were measured using a 500 MHz Bruker Avance Ultrashield spectrometer, with dimethyl sulfoxide (DMSO)‐d_6_ as solvent. Spectral processing and analysis was carried out using MestReNova software, with tetramethylsilane as the internal standard.

### 4.7 Magnetometry

Magnetic hysteresis loops were recorded on dried nanoparticle powders, using a Microsense EZ9 vibrating sample magnetometer. To mitigate geometric effects on the hysteresis loops, sample preparation was undertaken by drop‐casting purified nanoparticles, suspended in chloroform, onto a filter paper substrate (8 mm diameter), followed by drying under a high vacuum. The acquired hysteresis loops were further corrected for the presence of non‐magnetic organic‐ligand amounts by compensating for the samples' organic weight content, which was determined by thermogravimetric analysis (TGA).

The temperature‐dependent magnetic measurements were performed using a Quantum Design Physical Property Measurement System (PPMS) (Quantum Design, San Diego, CA, USA) equipped with a 9 T superconducting magnet and a vibrating sample magnetometer (VSM) option. The temperature dependence of the magnetization, (M(T)), was measured following the zero‐field‐cooled (ZFC) and field‐cooled (FC) protocols under an applied field of 50 Oe over the temperature range 5–300 K. In the ZFC protocol, the sample was first centered in the VSM under an applied field and then demagnetized to nominal zero field. The sample was subsequently cooled to 5 K in zero field without data acquisition. After reaching 5 K, a magnetic field of 50 Oe was applied, and the ZFC (M(T)) curve was recorded upon heating from 5 to 300 K at a rate of 5 K/min, with a temperature step of 1 K (i.e., one data point per 1 K). In the FC protocol, the measurement was conducted immediately after the ZFC run by cooling the sample from 300 to 5 K under the same applied field (50 Oe) at 5 K/min, while recording the magnetization with a 1 K temperature step.

### 4.8 Thermogravimetric Analysis (TGA)

TGA measurements on dried nanoparticle powders were conducted using a Mettler Toledo TGA/DSC 3+ Star System under steady O_2_ flow of 80 mL min^−1^, with a heating rate of 10 K min^−1^ over a temperature range from 30°C to 900°C.

### 4.9 Transmission Mössbauer Spectroscopy (TMS)

Mössbauer spectra were obtained in transmission mode at room temperature and pressure using a conventional spectrometer with constant acceleration with a 25 mCi ^57^Co radioactive source in a Rh matrix. The spectra were recorded in a standard multichannel analyzer using a velocity range of ±12.2 mm/s, and were subsequently fitted with the NORMOS software⁠. In all cases the area expressed in % corresponds to the fraction of Fe atoms in a particular environment with respect to the total amount of Fe atoms and the magnitudes inside parentheses are the standard deviation.

### 4.10 X‐Ray Diffraction (XRD) Analysis

The crystal structure of dried nanoparticle powders was analysed using a Malvern Panalytical Empyrean diffractometer (Malvern Panalytical GmbH, Kassel, Germany), which was equipped with a copper X‐ray source (λ = 1.5406 Å) and a PIXcel detector. Measurements were performed with a sweep rate of 0.2 s per step in the range of 4°≤ 2θ≤ 80°, with a step size of 0.04°. Curve processing and subsequent Rietveld refinement between 25°≤ 2θ≤ 60° was undertaken using Profex software.

### 4.11 Dynamic Light Scattering (DLS)

The particles' hydrodynamic diameters were measured with a Malvern Anton Paar Litesizer 500 DLS (Anton Paar Group AG, Graz, Austria) on re‐dispersed as‐synthesized nanoparticles in chloroform after purification.

### 4.12 Small Angle X‐Ray Scattering (SAXS)

Small‐angle X‐ray scattering (SAXS) measurements were performed using a Xenocs Xeuss 2.0 system. The system was equipped with a microfocus X‐ray source equipped with Cu target (λ = 0.154 nm, 8 keV). A Pilatus3R 200K‐A detector was used to record two‐dimensional scattering patterns. The sample‐to‐detector distance was set to 2400 mm. The sample was loaded in the liquid state into a glass capillary (Hilgenberg, 1.5 mm inner diameter). Measurements were conducted in a static mode with an exposure time of 300 s per frame. The corresponding time resolution for data acquisition was 0.1 s. The recorded 2D scattering patterns were azimuthally integrated to obtain one‐dimensional intensity profiles I versus 2θ. Data reduction and preliminary analysis were performed using the Xenocs XSACT software suite.

## Author Contributions

**Mingchen Ma**: methodology, Writing – review and editing, investigation. **Pu Luo**: methodology, investigation, writing – review and editing. **Lukas Hertle**: methodology, conceptualization, data curation, investigation, validation, formal analysis, visualization, writing – original draft, writing – review and editing. **Chenyi Zhou**: investigation, methodology, formal analysis, validation. **Chuanyi Wu**: methodology, resources, investigation. **Chen Chen**: methodology, investigation. **Chenxin Gao**: methodology, resources, investigation. **Fei Chen**: methodology, resources, investigation. **Ziyu Li**: methodology, investigation, writing – review and editing. **Xiang‐Zhong Chen**: conceptualization, methodology, supervision, formal analysis, funding acquisition, project administration, resources, writing – review and editing, writing – original draft. **Joaquim Llacer‐wintle**: methodology, writing – original draft, writing – review and editing, data curation, investigation. **Valentin Gantenbein**: methodology, writing – original draft, writing – review and editing, investigation. **Carlos Franco**: methodology, data curation, investigation, writing – original draft, writing – review and editing. **Josep Puigmartí‐luis**: writing – original draft, methodology, investigation. **Pere Bruna**: methodology, data curation, investigation, formal analysis, writing – original draft. **Zhengwei Tan**: methodology, investigation, writing – review and editing. **Salvador Pané**: supervision, funding acquisition, project administration, resources, writing – original draft, writing – review and editing, conceptualization, methodology. **Bradley J. Nelson**: resources, supervision, project administration, writing – original draft, writing – review and editing.

## Funding

This work is supported by the National Science Foundation of China (Project No. 52473254, 52503312), Science and Technology Commission of Shanghai Municipality (24520750200), Shanghai Talent Programs,Zhejiang Provincial Postdoctoral Research Project First‐Class Funding, China, (Project No. ZJ2024029), Swiss National Science Foundation (Project No. 198643, and 190451), Grant PID2023–146623NB‐I00 funded by MICIU/AEI/10.13039/501100011033/FEDER/UE, Generalitat de Catalunya AGAUR grant 2021‐SGR‐00343, Maria de Maeztu Units of Excellence Programme CEX2023–001300‐M / funded by MCIN/AEI / 10.13039/501100011033.

## Conflicts of Interest

The authors declare no conflicts of interest.

## Supporting information




**Supporting File**: advs76632‐sup‐0001‐SuppMat.pdf.

## Data Availability

The data that support the findings of this study are available from the corresponding author upon reasonable request.
