# Supplementary material for: Solvent Oxidation‐Directed Synthesis of Highly Crystalline Iron Oxide Nanoparticles With Near‐Bulk Magnetization
Source: Adv Sci (Weinh). 2026 Jul 20;13(56):e76632. doi: 10.1002/advs.76632 (PMC13383684; doi:10.1002/advs.76632)
Supplement: Supplementary file 1 [file ADVS-13-e76632-s001.pdf]

Supporting Information

Solvent Oxidation-Directed synthesis of Highly Crystalline Iron Oxide Nanoparticles with Near-Bulk Magnetization

Lukas Hertle, Valentin Gantenbein, Joaquim Llacer-Wintle, Mingchen Ma, Ziyu Li, Pu Luo, Zhengwei Tan, Fei Chen, Chen Chen, Chuanyi Wu, Chenxin Gao, Chenyi Zhou, Carlos Franco, Pere Bruna, Josep Puigmartí-Luis, Xiang-Zhong Chen*, Bradley J. Nelson, Salvador Pané**

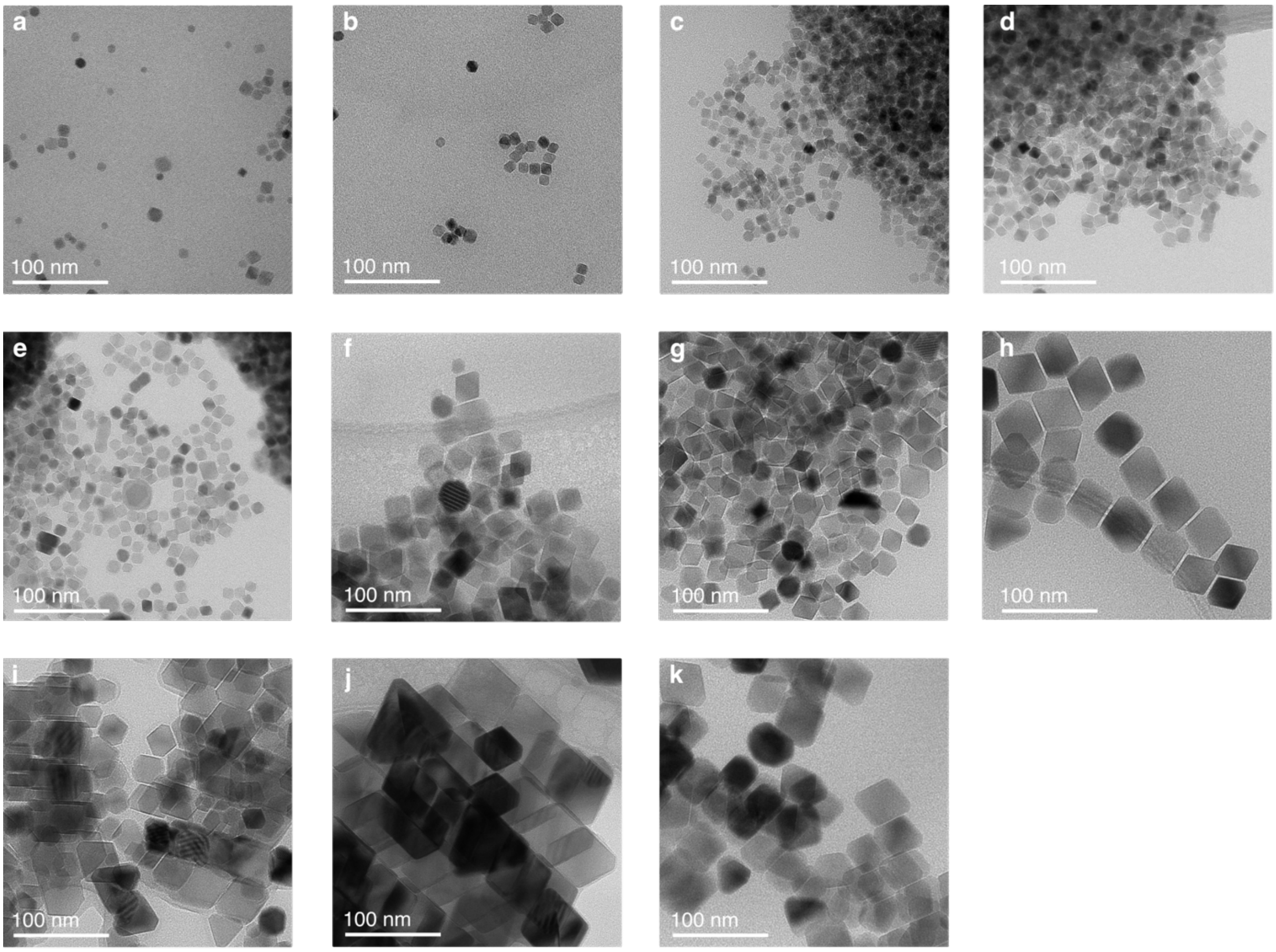

Figure S1. TEM images of iron oxide nanoparticles synthesized with benzyl ether oxidized for different time durations. a) 0 hours. b) 5 hours. c) 10 hours. d) 15 hours. e) 20 hours. f) 25 hours. g) 30 hours. h) 35 hours. i) 40 hours. j) 45 hours. k) 50 hours.

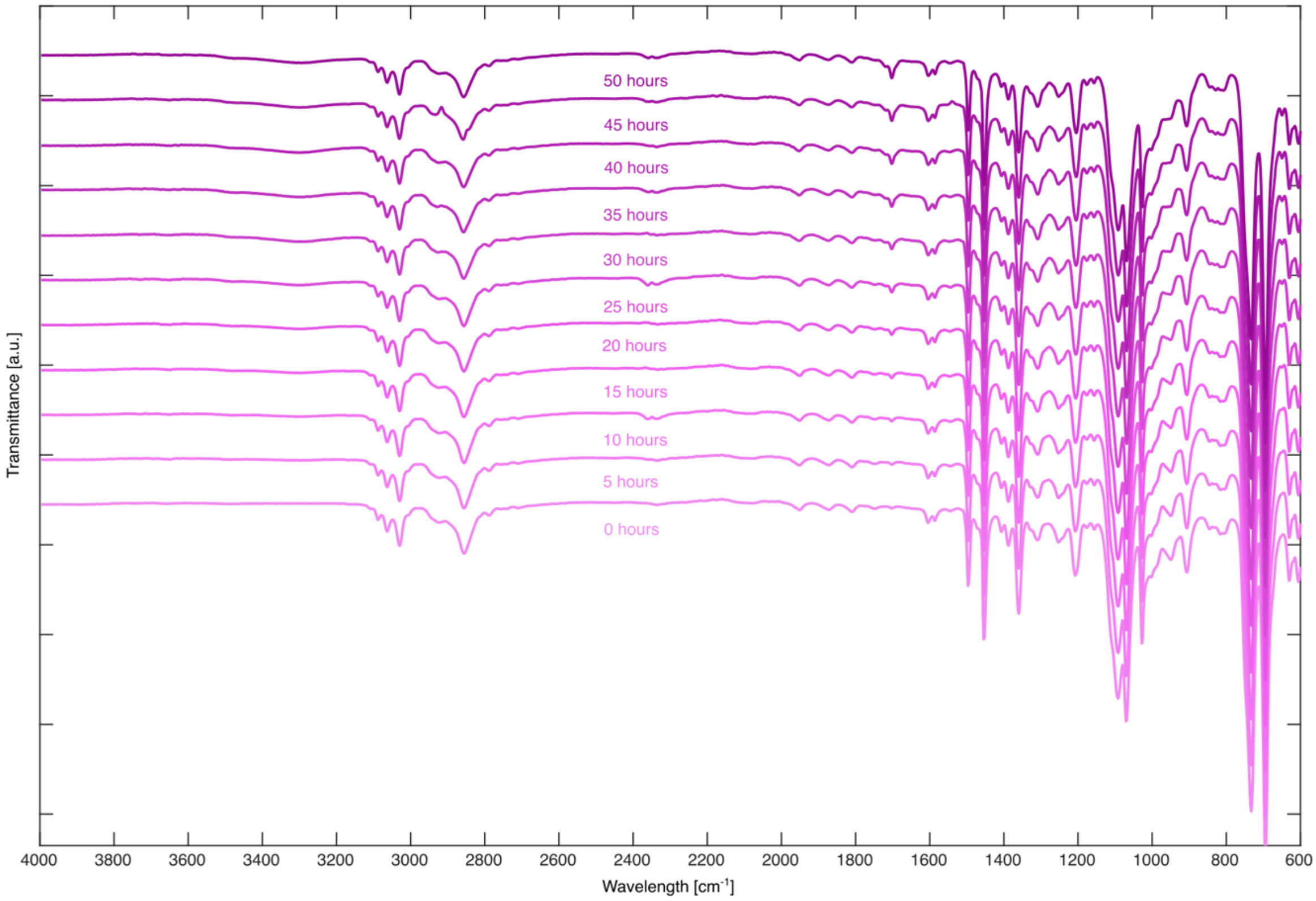

Figure S2. FTIR spectra of benzyl ether oxidized for different time durations

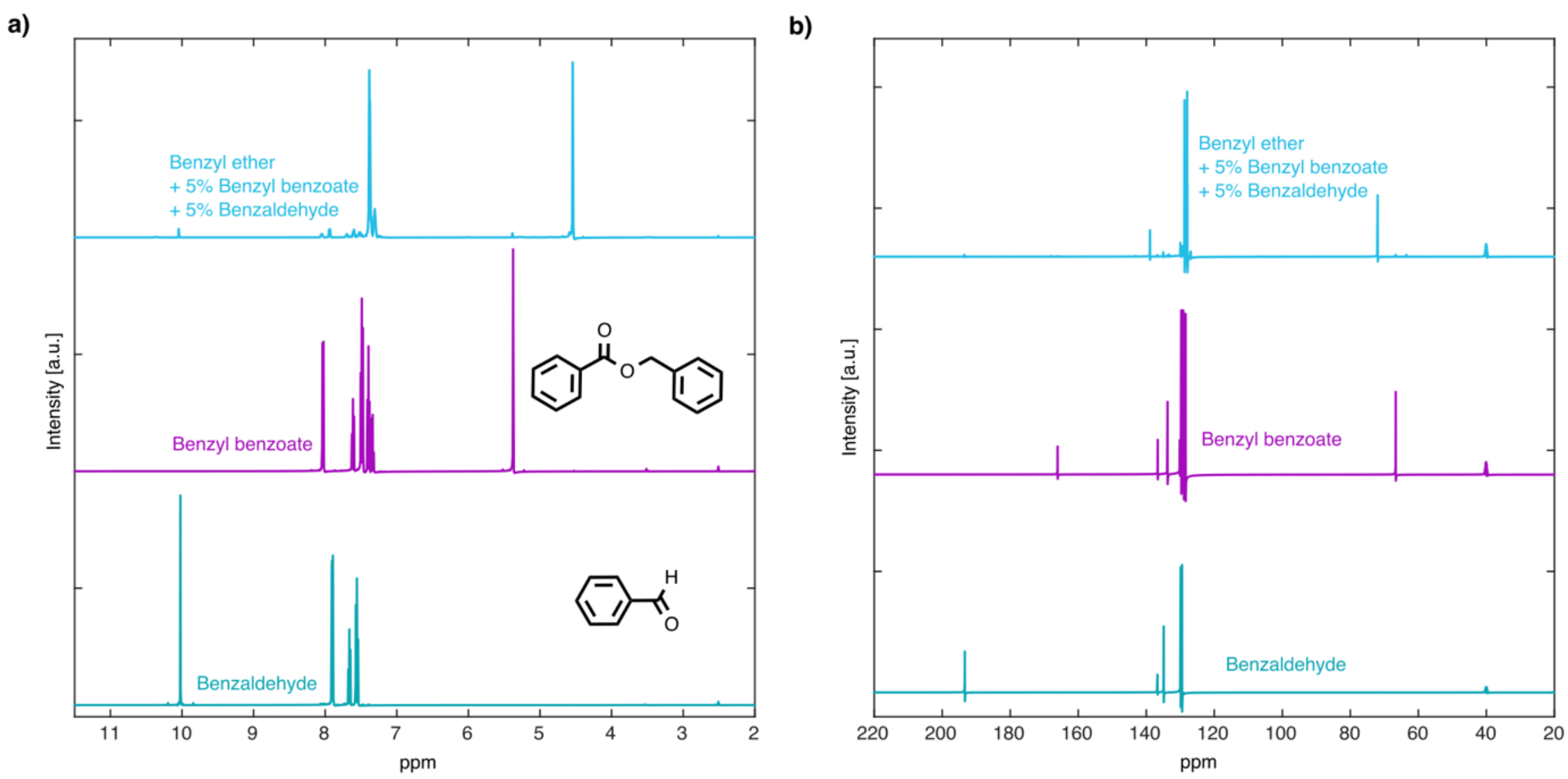

Figure S3. NMR spectra of benzaldehyde, benzyl benzoate, and pristine benzyl ether spiked with 5% of each. a) ^{1}H NMR. b) ^{13}C NMR

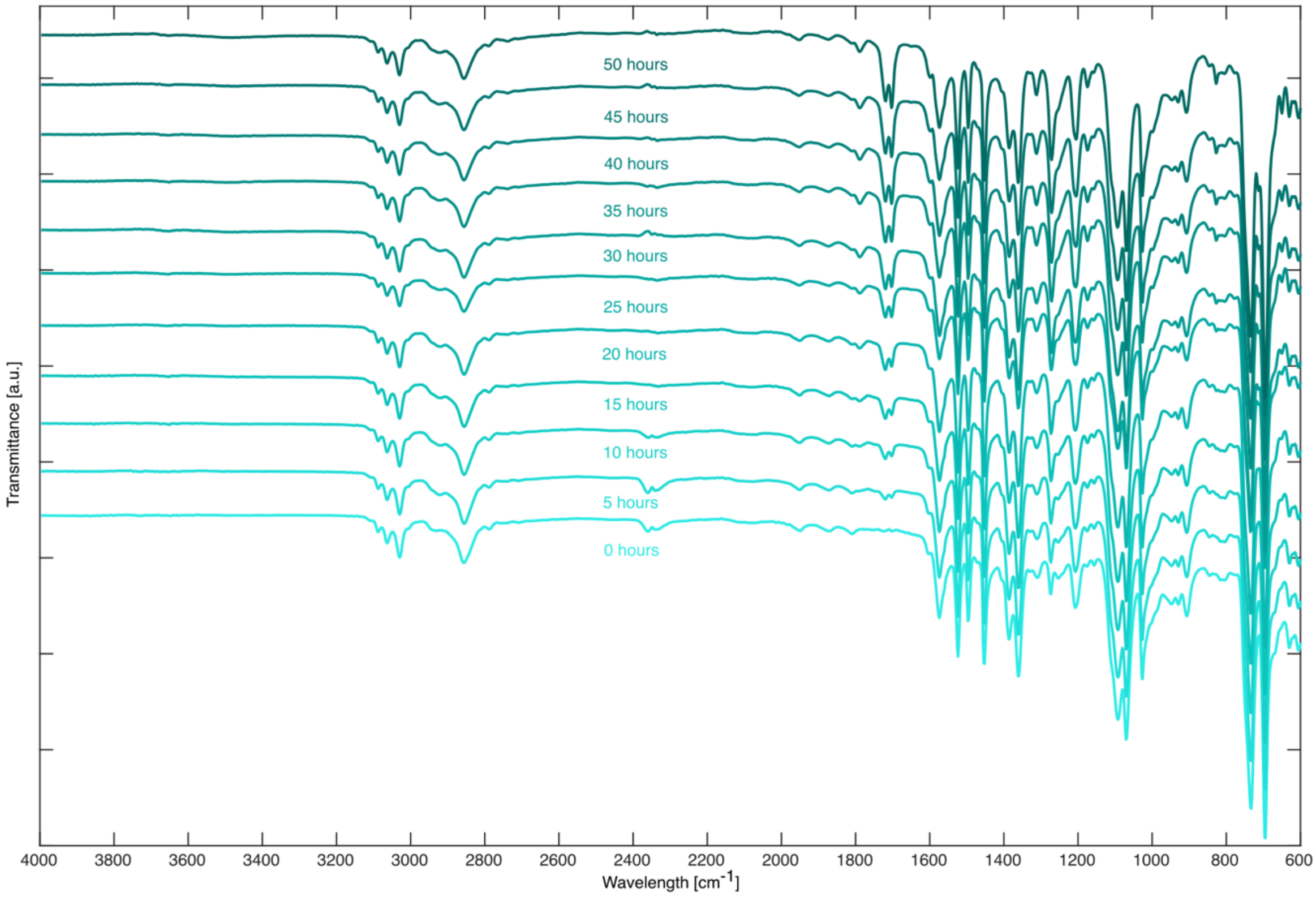

Figure S4. FTIR spectra of benzyl ether oxidized for different time durations mixed with $Fe(acac)_3$

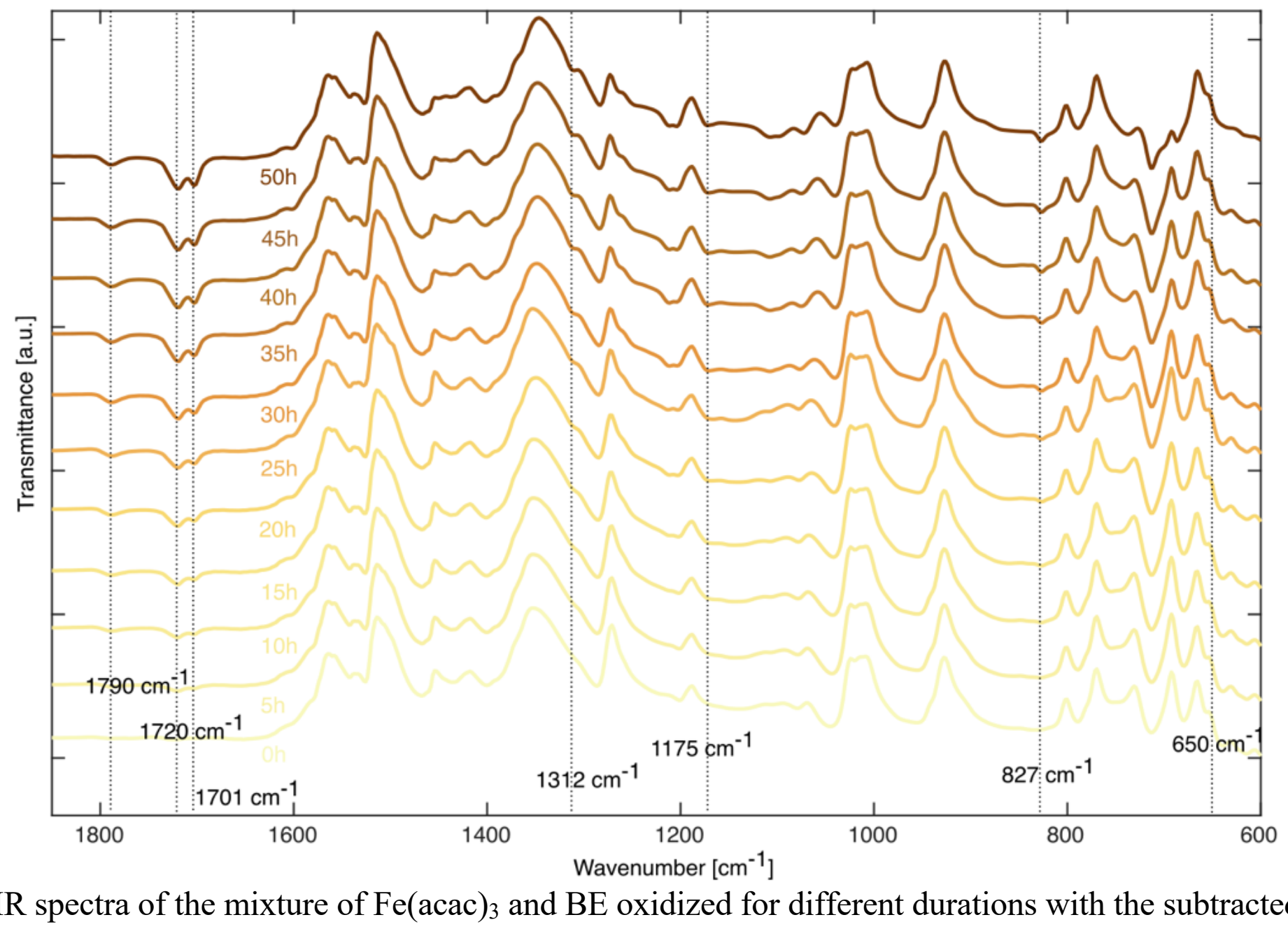

Figure S5. FTIR spectra of the mixture of $Fe(acac)_3$ and BE oxidized for different durations with the subtracted individual spectra of $Fe(acac)_3$ and oxidized BE

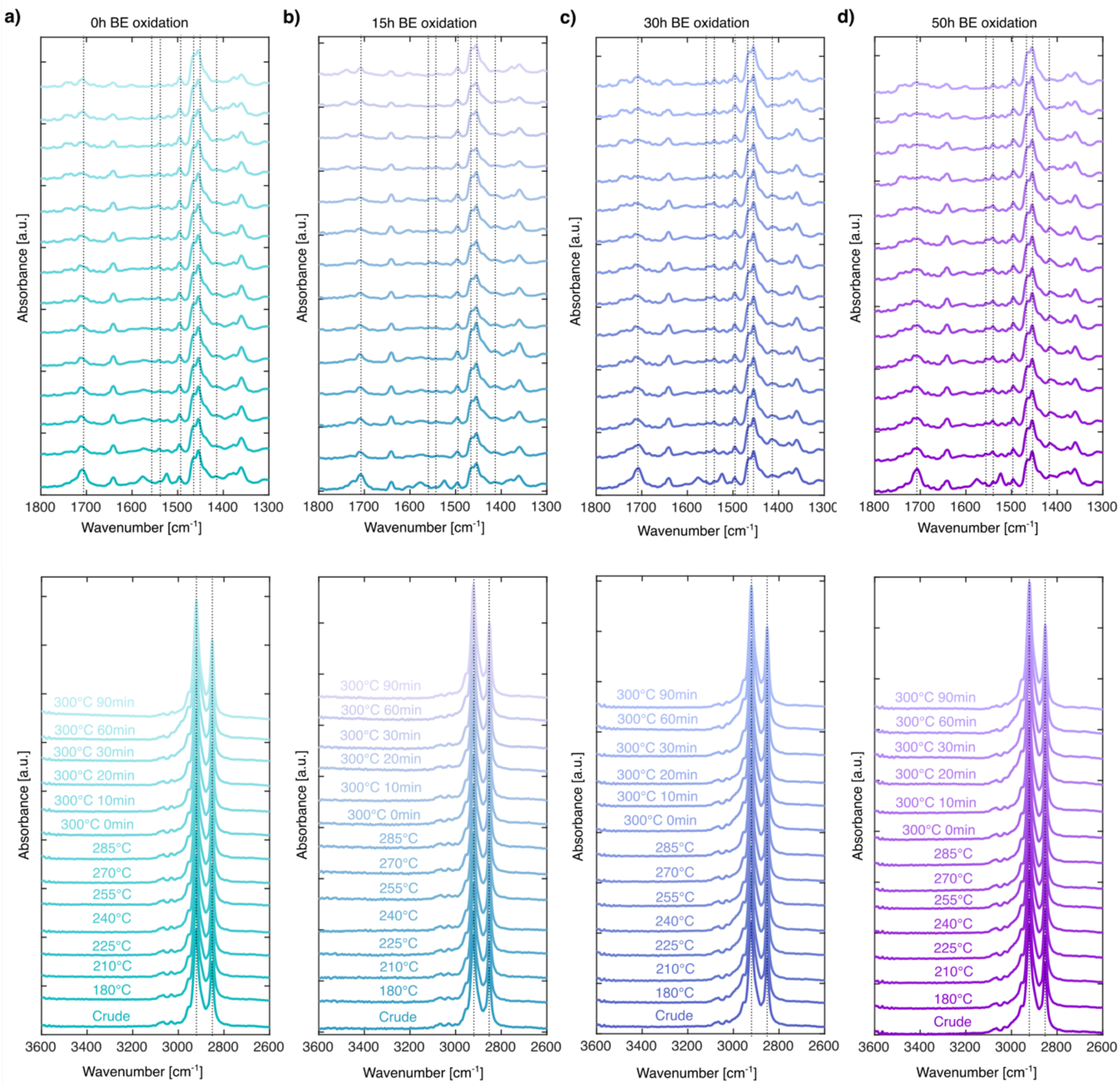

Figure S6. FTIR spectra of aliquotes taken throughout the synthesis of the nanoparticles with benzyl ether oxidized for different duration. a) 0 hours. b) 15 hours. c) 30 hours. d) 50 hours.

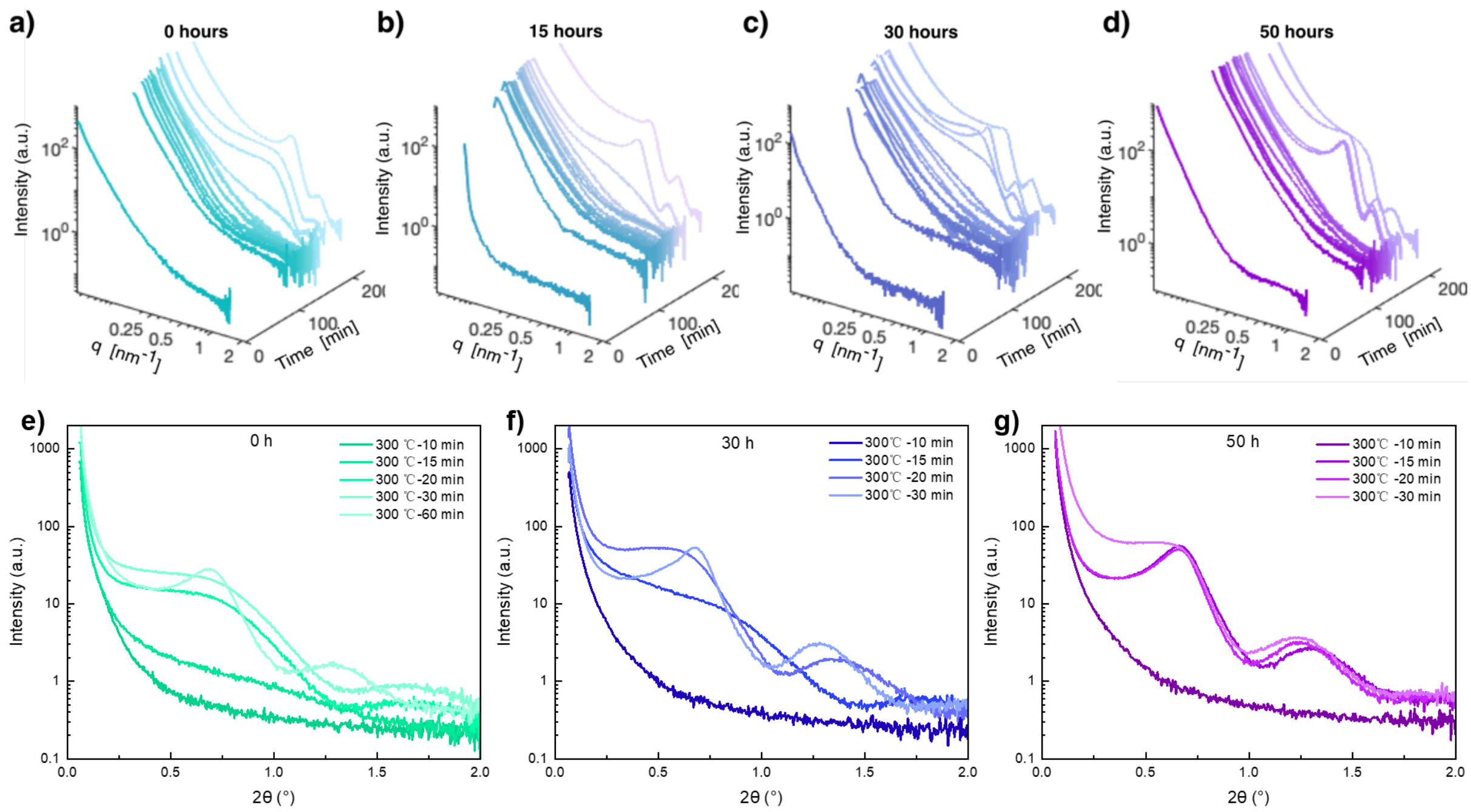

Figure S7. SAXS characterization of nanoparticle structural evolution. (a–d) Overview of SAXS profiles for sample aliquots collected throughout the synthesis using BE oxidized for 0 h, 15 h, 30 h, and 50 h, respectively. (e–g) Magnified views of the SAXS curves at specific time intervals after reaching the 300 °C plateau for the (e) 0 h, (f) 30 h, and (g) 50 h samples.

We performed ex situ small-angle X-ray scattering (SAXS) analysis on the collected sample aliquots, with an overview of the resulting profiles for all groups presented in Figure S7 (a–d). It can be seen more clearly from the magnified profiles in Figure S7(e–g) that the scattering profiles exhibited a pronounced increase in intensity approximately 15 minutes after reaching 300 °C, marking the nanoparticle formation. Intriguingly, these time points are highly congruent with the interval where the intensity ratio of the IR peaks at 1734 cm^{-1} and 1708 cm^{-1} reaches its maximum (Figure 5f), suggesting that the observed spectral evolution is intrinsically linked to the particle formation event. Beyond the overall rise in intensity, the emergence of well-defined scattering peaks over time signifies the development of highly monodisperse nanoparticles and their subsequent correlated arrangements. This is a characteristic signature of uniform crystalline nanoparticle formation, as previously reported.[1] Furthermore, the rate of intensity increase was notably accelerated with prolonged BE oxidation times, with peaks appearing at earlier reaction stages, which are consistent with enhanced nanoparticle growth kinetics.

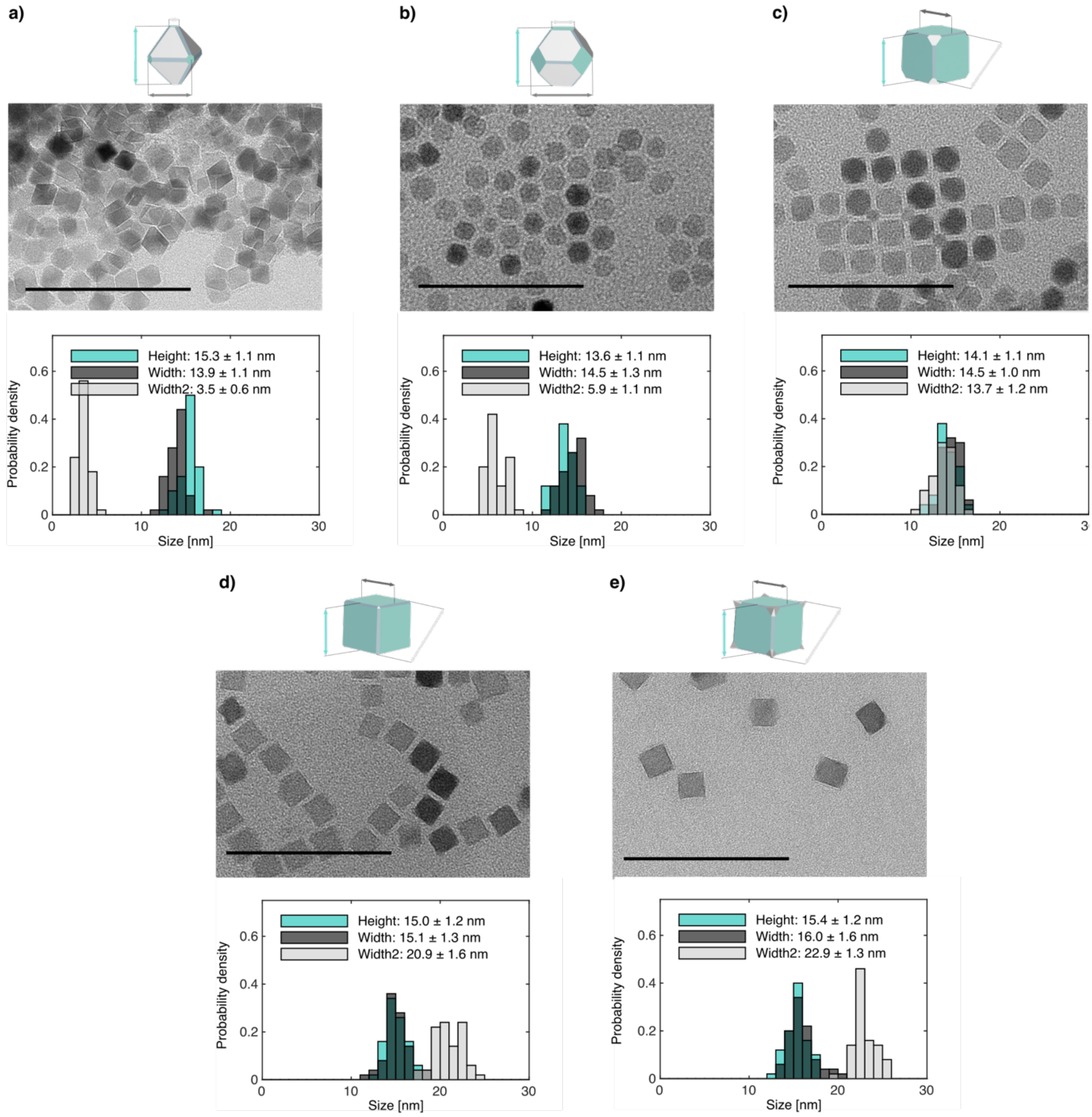

Figure S8. TEM images of iron oxide nanoparticles synthesized with benzyl ether oxidized for 15 hours, while changing the molar ratio between oleic acid to sodium oleate (OA:SO) in the reaction: a) 100:0, b) 97.5:2.5, c) 95:5, d) 92.5:7.5, e) 90:10.

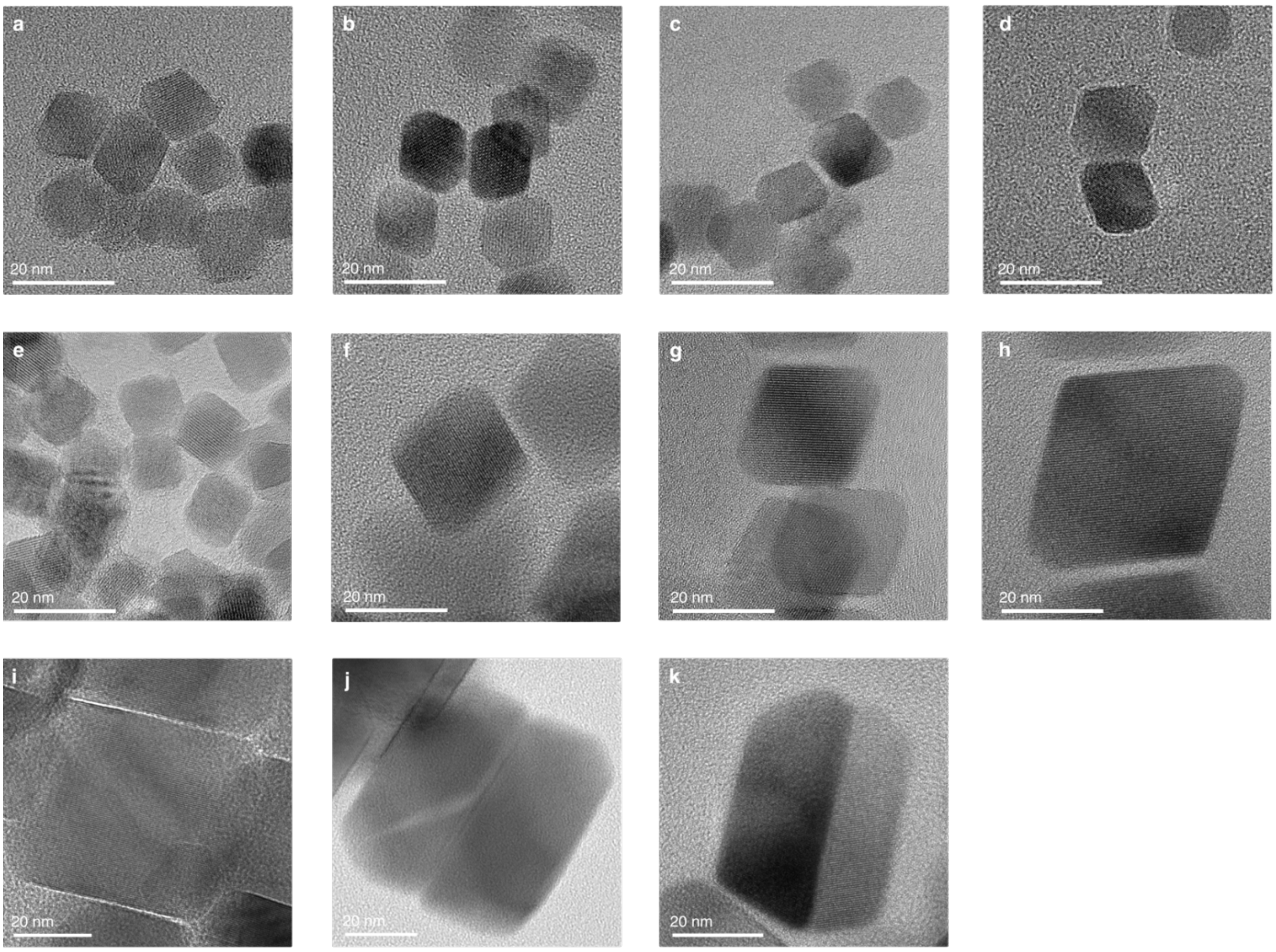

Figure S9. HRTEM images of iron oxide nanoparticles synthesized with benzyl ether oxidized for different time durations. a) 0 hours. b) 5 hours. c) 10 hours. d) 15 hours. e) 20 hours. f) 25 hours. g) 30 hours. h) 35 hours. i) 40 hours. j) 45 hours. k) 50 hours.

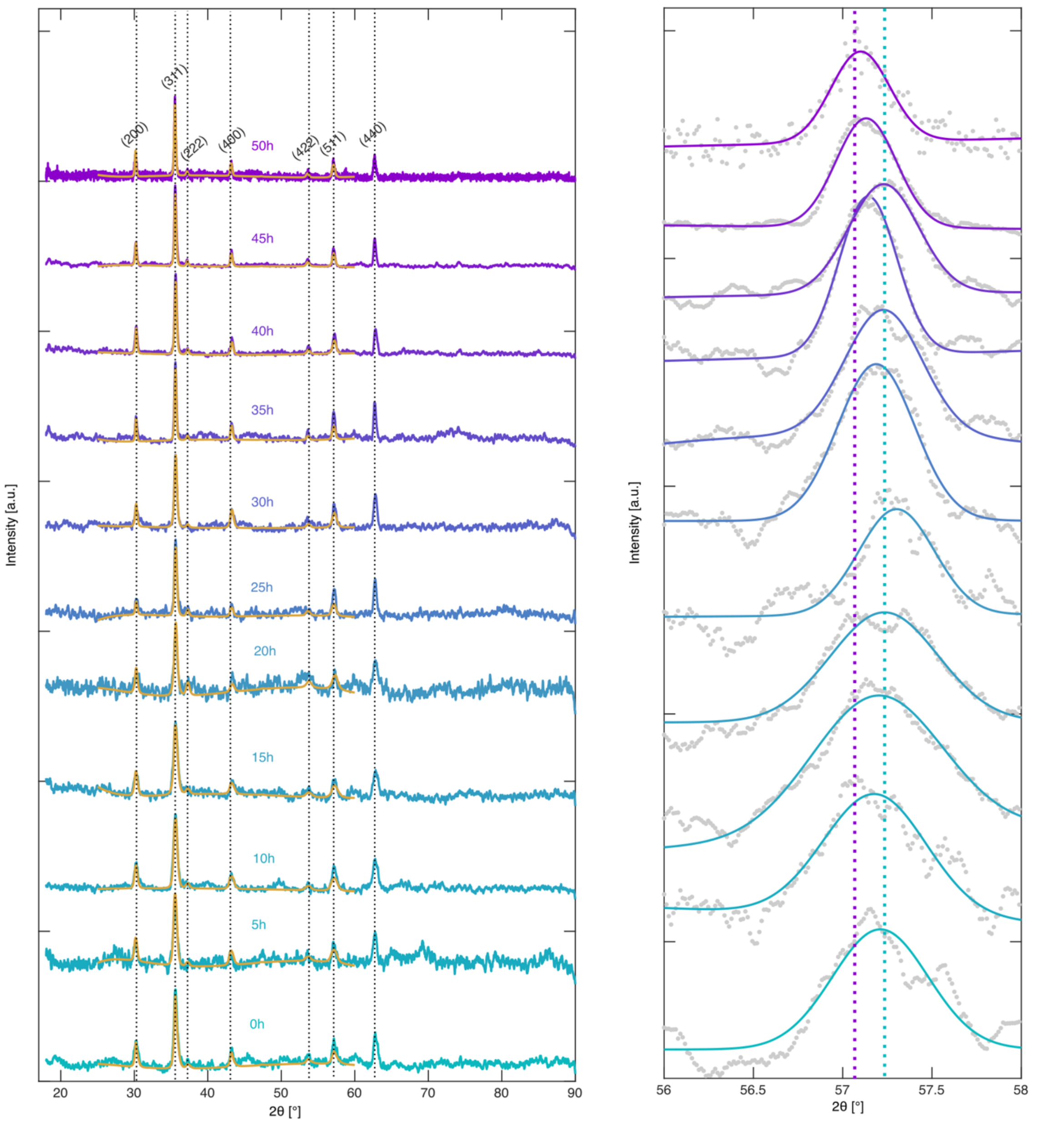

Figure S10. a) XRD patterns of particles synthesized with benzyl ether oxidized for different oxidation times with Rietveld refinement fitted curve (orange). b) (511) plane associated peaks of the spectra with their respective gaussian fitting curves.

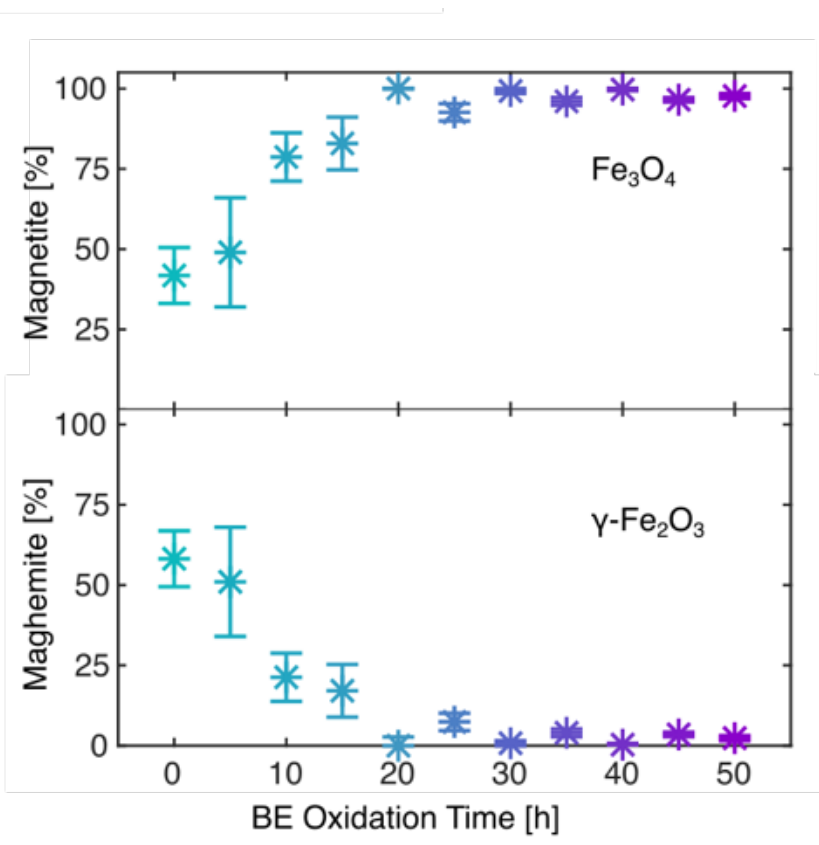

Figure S11. Phase percentage calculated from Rietveld refinement for particles synthesized with benzyl ether oxidized for different oxidation times

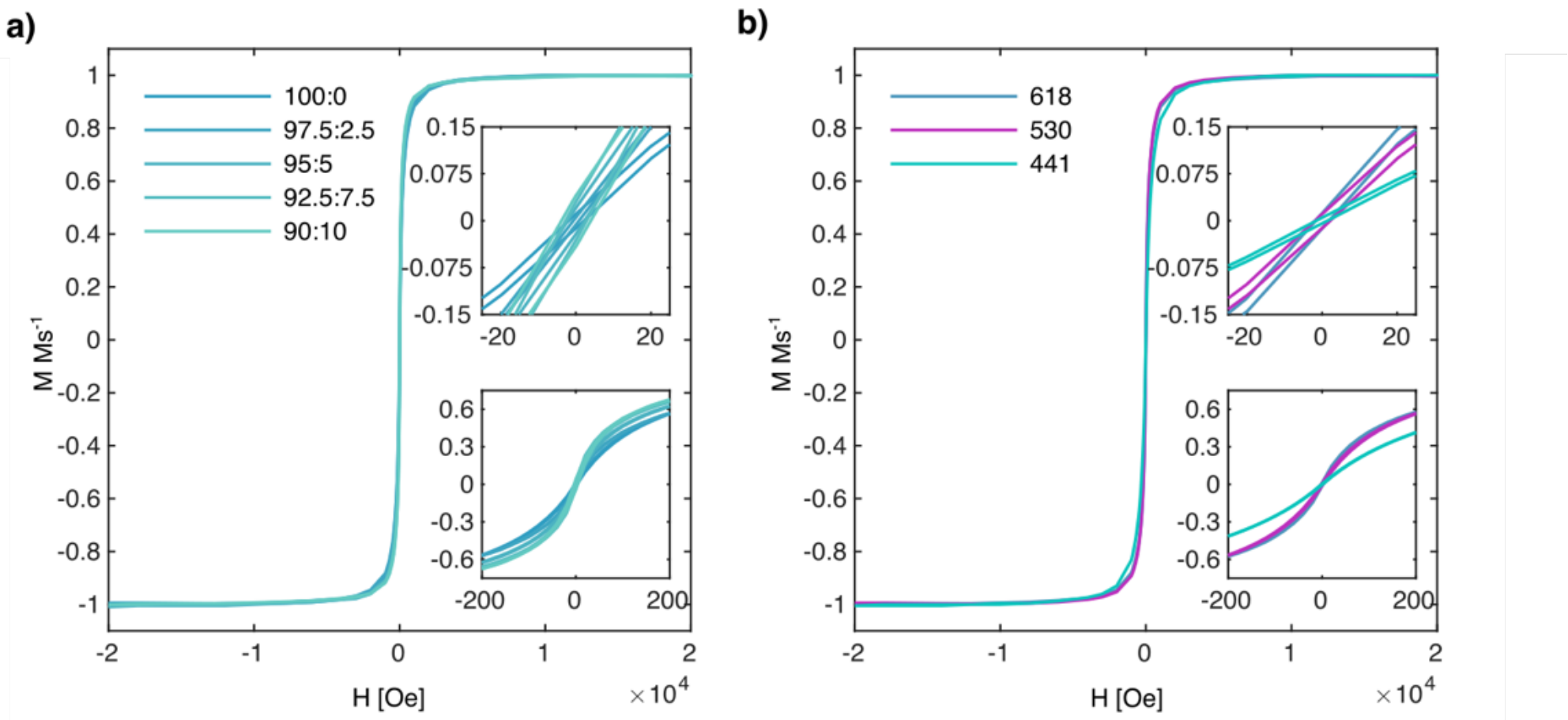

Figure S12. Normalized hysteresis loops of: a) Morphology controlled nanoparticles by adjusted OA:SO ratios. b) Size controlled particles by the adjustment of the utilized precursor amounts.

0h

	Fe^{3+}	$Fe^{2.5+}$	Distr.	SP
**BHF(T)**	46.4 (1)	41.6 (2)	24.0 (8)	-
**δ (mm/s)**	0.45 (1)	0.52 (1)	0.67 (3)	0.24 (6)
**Δ (mm/s)**	0.0	0.005	0.18 (3)	0.4 (1)

15h

	Fe^{3+}	$Fe^{2.5+}$	Distr.	SP
**BHF(T)**	46.1 (1)	40.9 (2)	24.4 (5)	-
**δ (mm/s)**	0.38 (1)	0.53 (1)	0.68 (3)	0.56 (6)
**Δ (mm/s)**	0.0	0.005	0.6 (3)	0.0 (1)

30h

	Fe^{3+}	$Fe^{2.5+}$
**BHF(T)**	48.6 (1)	45.4 (2)
**δ (mm/s)**	0.29 (1)	0.63 (1)
**Δ (mm/s)**	0.005 (3)	-0.004 (4)

50h

	Fe^{3+}	$Fe^{2.5+}$
**BHF(T)**	49.2 (1)	45.9 (1)
**δ (mm/s)**	0.28 (1)	0.65 (1)
**Δ (mm/s)**	0.00 (2)	0.004 (2)

Table S1. Hyperfine parameters: isomer shift (δ), expressed relative to the isomer shift of the bcc-Fe at room temperature, the quadrupole splitting (Δ) and the hyperfine magnetic field (BHF). For the distribution (Distr.), the hyperfine magnetic field is the average value. SP stands for the superparamagnetic contribution.

References

[1] J. Zhang, S. Werner, A. Köppen, X. Liu, C. Shen, F. Bertram, W. J. Parak, X. Sun, "Unraveling the synthesis and assembly of gold-iron oxide hybrid nanoparticles" *Nano Today* **2024**, *57*, DOI 10.1016/j.nantod.2024.102384.
